# IncC helper dependent plasmid-like replication of Salmonella Genomic Island 1

**DOI:** 10.1093/nar/gkaa1257

**Published:** 2021-01-06

**Authors:** Mónika Szabó, Gábor Murányi, János Kiss

**Affiliations:** Agricultural Biotechnology Institute, National Agricultural Research and Innovation Centre, Gödöllő H2100, Hungary; Agricultural Biotechnology Institute, National Agricultural Research and Innovation Centre, Gödöllő H2100, Hungary; Agricultural Biotechnology Institute, National Agricultural Research and Innovation Centre, Gödöllő H2100, Hungary

## Abstract

The *Salmonella* genomic island 1 (SGI1) and its variants are mobilized by IncA and IncC conjugative plasmids. SGI1-family elements and their helper plasmids are effective transporters of multidrug resistance determinants. SGI1 exploits the transfer apparatus of the helper plasmid and hijacks its activator complex, AcaCD, to trigger the expression of several SGI1 genes. In this way, SGI1 times its excision from the chromosome to the helper entry and expresses mating pore components that enhance SGI1 transfer. The SGI1-encoded T4SS components and the FlhDC-family activator proved to be interchangeable with their IncC-encoded homologs, indicating multiple interactions between SGI1 and its helpers. As a new aspect of this crosstalk, we report here the helper-induced replication of SGI1, which requires both activators, AcaCD and FlhDC_SGI1_, and significantly increases the stability of SGI1 when coexists with the helper plasmid. We have identified the *oriV*_SGI1_ and shown that *S004-repA* operon encodes for a translationally coupled leader protein and an IncN2/N3-related RepA that are expressed under the control of the AcaCD-responsive promoter P*_S004_*. This replicon transiently maintains SGI1 as a 4–8-copy plasmid, not only stabilizing the island but also contributing to the fast displacement of the helper plasmid.

## INTRODUCTION

Integrative mobilizable elements (IMEs) are prevalent vehicles of the antibiotic resistance and virulence factors in bacteria (1). More than 250 IMEs have been identified to date, occuring in a wide range of Gram− and Gram+ species (1,2). Unlike integrative conjugative elements (ICEs, formerly known as conjugative transposons (3)), IMEs devoid of the complete conjugation gene set. To ensure their horizontal spread, they have to hijack the conjugative apparatus of other elements, such as conjugative plasmids or ICEs, (4). Since IMEs cannot conjugate autonomously, they apply many different strategies to exploit their helper elements for mobilization (5). After the entry into a recipient cell, IMEs (like ICEs) integrate into the host chromosome, ensuring their stable vertical transmission. Integration is an autonomous function fulfilled by Tyr or Ser site-specific recombinases or even by DDE transposases. The elements with Tyr recombinases often target the 3′ end of tRNA genes or other conserved genes, while members of the other two groups show lower target specificity (1).

**Figure 1.**
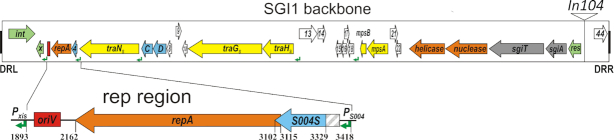
Schematic map of conserved SGI1 backbone. The annotated ORFs *S001-S044* are indicated by colour-coded arrows: green – recombinase, transposase; orange – replication; blue – transcription regulator; yellow – conjugation; grey – TA system; white – unknown function. Names of genes with known function or identified homologs are indicated. box. Abbreviations: *x* − *xis*, *C* and *D* − *flhC*_SGI1_ and *flhD*_SGI1_, *sgiT* and *sgiA* – toxin and antitoxin genes of the TA system. Terminal direct repeats are shown as black boxes, the position of In104 is indicated. AcaCD-responsive promoters are shown as green angled arrows. The *oriV* identified in this work is depicted by a red box. ORFs of interest in this work are shown in a magnified interval below. The untranslated 5′ part of *S004* is hatched. Coordinates are given according to the excised SGI1 sequence generated by deleting the first 145 bp and the last 5145 bp from the published SGI1 sequence (GenBank: AF261825).

SGI1 is a founder element of a large IME family that was identified in a multidrug resistant (MDR) clone of *Salmonella enterica* serovar Typhimurium DT104 (6) in the mid-1980s (7). This pandemic clone spread worldwide, possibly due to the resistance to many antibiotics (ACSSuT phenotype) conferred by the 42.4-kb island. SGI1 and most of its variants identified in numerous *S*. *enterica* serovars and *Proteus mirabilis* strains (8–12) share a conserved backbone consisting of an integration/excision (*int* and *xis*) module, a *rep* gene encoding a putative replication initiation protein, genes related to plasmid-borne T4SS genes (*traN, traG, traH*) (13), a pair of genes encoding FlhDC-family regulators (14), a mobilization module with the transfer origin (*oriT*) (15), a helicase and a nuclease gene (13), a TA system (16), a resolvase gene (13) and several further ORFs with unknown functions (*S004, S008-S010, S013-S018, S044*, Figure 1). In most SGI1 variants the backbone is interrupted between the *res* and *S044* genes by a complex In4-type integron structure named In104, which contains the resistance genes, IS elements (IS*CR3*, IS*6100*), and some additional genes of unknown function (13). Most of the variants differ only in the resistance genes that they harbour in the In104 cluster, however, more divergent SGI1-relatives such as PGI1, PGI2, AGI1 (12,17,18) show larger sequence divergence also in their backbones (15).

SGI1-family islands integrate into or excise from the host chromosome by a site-specific recombination at the 3′-end of *trmE* gene (also called as *thdF* or *mnmE*). The reaction is carried out by the self-encoded λ-integrase-family recombinase Int and the recombination directionality factor (RDF), Xis. The excised circular form of the island can be mobilized exclusively by the broad host range conjugative IncA and IncC helper plasmids (19,20), whose conjugative system is classified into the MOB_H12_ group (21). The conjugation apparatus and several other genes of these plasmids are controlled by the FlhDC-family activator complex, AcaCD (22), which is essential for the plasmid transfer and the mobilization of SGI1 (14). In addition to the conjugation genes of the plasmid, AcaCD also activates Xis expression on SGI1 leading to the excision of the island (14,22). In absence of the helper plasmid, SGI1 is very stable (23) although spontaneous excision is still detectable by nested PCR (19). This stability is probably based on the low excision activity of the constitutively expressed Int (14) and the very low level of Xis (24). In contrast, a high rate of SGI1 loss has been detected in the presence of the helper plasmid due to the AcaCD-dependent triggering of excision (14). This effect is reduced by the TA system of the island (16), however, it cannot completely stabilize the co-habitation of SGI1 and the helper plasmid (14). Furthermore, SGI1 destabilizes the IncA and IncC plasmids (16,25) by a yet unknown manner, which might also contribute to the long-term stability of the island. The transient replication can also increase the stability of the excised element. Relaxase-dependent rolling circle replication in donor cells appears to be general among SXT/R391-family (26) and some other ICEs, like ICE*Bs1*, ICE*St3* and Tn*916* (27). This type of replication begins at the nick site of *oriT* used as an origin of replication (*oriV*) and relies on the relaxase acting as the replication initiator (28). In the case of ICE*Bs1*, this conjugation-associated transient replication is required for the stability the ICE (29). This sigma-type or rolling-circle replication may be an inherent feature of the conjugation module of ICEs, which can contribute to their stable maintenance. Another way of replication is a circle-to-circle or theta-replication, which is common in many plasmid families and has been shown to occur also in several ICEs. For example, theta-replication ensures the stability of the extrachromosomal forms of Tn*GBS1* and Tn*GBS2* from *S. agalactiae* (30). Regarding IMEs, similar replication mechanisms have not yet been reported, even though many IMEs encode for relaxases and proteins related to replication initiator proteins of RepA, RepC or Rep_3 families (5). SGI1 also encodes for a Rep_3 family Rep protein (S003), but the cognate *oriV* has not been identified previously (31), and the island has been thought to be a non-replicable element (19).

SGI1 not only stabilizes itself in various ways when the helper plasmid enters, but enhances the self-transfer rate by remodelling its conjugation apparatus by the AcaCD-induced expression of TraN_S_, TraH_S_ and TraG_S_. These SGI1 proteins are homologous to and can replace the TraN_C_, TraH_C_ and TraG_C_ of the IncC plasmids and ensure some advantages for SGI1 transfer at the expense of the helper (32). Similar compatibility has been shown between AcaCD and the closely related SGI1-encoded regulator, FlhDC_SGI1_ (also known as SgaCD). The latter is able to rescue the transfer-deficiency of the *acaCD*-deleted IncC plasmid and activate the AcaCD-controlled promoters of SGI1 (33), however, the exact function of FlhDC_SGI1_ is not yet clear. In addition to *xis*, *traN* and the *traGH* operon, the ORFs *S004* and *S018* are also controlled by AcaCD-responsive promoters, but the functions of the putative proteins encoded are unknown. However, S004 protein, which proved to be translated from an inner START codon of ORF *S004* (and named as S004S), has some negative effects on the bacterial growth (33).

As a new aspect of the complex crosstalk between SGI1 and IncC plasmids, we report here the plasmid-like replication of SGI1, which requires both activators, AcaCD and FlhDC_SGI1_. This is the first example of a helper-induced replication of an IME. We have identified the *oriV* and show that the Rep protein encoded by ORF *S003* is related to RepA of IncN2 plasmids. The ORF *S004* overlapping with *repA* encodes for a leader peptide and its translation is essential for RepA expression. The AcaCD-induced replication results in 4–8 circular plasmid-like SGI1 copies per cell, which is indispensable for stable maintenance when the helper plasmid is present and may also contribute to the fast elimination of the helper and the high frequency of SGI1 transfer.

## MATERIALS AND METHODS

### DNA and microbial techniques

Standard molecular biology procedures were carried out according to (34). For comparison of the amount of excised SGI1 with or without excess RepA, plasmid DNA was isolated from 1.5 ml overnight cultures of TG1Nal::SGI1-C/R55^ΔTn^*^6187^* strain +/− pGMY9 (RepA producer plasmid) using the alkaline lysis method. Total DNA was prepared as described in (35). Enzymes were purchased from Thermo Fisher Scientific, New England Biolabs and Roche, chemicals are from Sigma, Roth and Reanal. Test/colony PCRs were performed using Dream Taq polymerase (Thermo Fisher Scientific) as described in (23). For cloning purposes, high-fidelity DNA polymerases Pwo (Roche) or Phusion (Thermo Scientific) were applied and the cloned PCR products were sequenced on ABI Prism 3100 Genetic Analyzer (Perkin Elmer). Oligonucleotides used in this work are listed in Supplementary Table S1. Primers were designed using the published sequence of R16a (GenBank: KX156773), R55 (GenBank: JQ010984) and the ‘excised’ SGI1 generated by deleting the first 145 bp and the last 5145 bp of the published SGI1 sequence (GenBank: AF261825).

Gene KO experiments were carried out by the one-step recombination method (36) using the λ Red recombinase producer plasmid pKD46 and pKD3 or pKD4 (where it is indicated) template plasmids for amplification of the KO fragments. For deletion of *oriV* with or without P*_xis_* in TG1Nal::SGI1-C, sgiPxisdelfor1-sgiPxisdelrev or sgiPxisdelfor2-sgiPxisdelrev primers were used to amplify the KO fragments, respectively. The full rep region along with P*_xis_* was deleted using sgiPxisdelfor1-SGI_S004–005delrev primer pairs resulting in SGI1-C^Δrep_region^. ORF *S003* (*repA*_SGI1_) was deleted using primers SGI_S003delfor-SGI_S003delrev resulting in SGI1-C^Δ^*^repA^*. For the construction of SGI1-C^ΔP^*^S004^*, SGI_S004–005delfor-SGI_S004–005delrev primers were used. The *flhDC* of SGI1-C^Δrep_region^ and the *acaCD* of R55^ΔTn^*^6187^*were deleted using the pKD4 template plasmid and sgi006delfor-sgi007delrev and delflhDCfor-delflhDCrev primer pairs, respectively. Electroporation of KO fragments was carried out using BTX Electro Cell Manipulator 600 with 2-mm gap electroporation cuvettes as described (35).

Bacterial strains listed in Supplementary Table S2 were routinely grown at 37°C in LB broth/agar supplemented with the appropriate antibiotics used at a final concentration as follows: ampicillin (Ap) 150 μg/ml, chloramphenicol (Cm) 20 μg/ml, kanamycin (Km) 30 μg/ml, spectinomycin (Sp) 50 μg/ml, streptomycin (Sm) 50 μg/ml, nalidixic acid (Nal) 20 μg/ml, gentamicin (Gm) 25 μg/ml, tetracycline (Tc) 10μg/ml. For maintaining and curing the plasmids with a temperature-sensitive pSC101 replication system 30 and 42°C were applied, respectively. Bacterial strains were maintained at −80°C in LB broth containing 30% glycerol. In conjugation-, stability-, and RT-qPCR-assays, the interchangeable IncC plasmid R16a or R55 was applied as helper depending on the required resistance markers in the particular experimental setup. Their equivalence in excision induction and mobilization of SGI1, as well as in their self-transfer rate has previously been shown (20,15). Matings were carried out as described previously (23).

The β-galactosidase assays (37) were performed in 4 replicates as described by (14). The activity of P*_S004_* promoter was measured using the β-galactosidase test plasmid pMSZ965 harbouring the upstream region of ORF *S004S*. To test the translation activity of RepA depending on the translation of S004 protein, the 3′ truncated *repA* gene was fused in-frame to the eighth codon of *lac*Z (38) in the pMSZ1030 derivative plasmids pMSZ1032, pMSZ1034, pMSZ1037, pMSZ1039 and the β-galactosidase activity of the resulting fusion proteins was measured.

For compatibility tests, pMSZ1012 (*oriV*_SGI1_+P_tac_::*repA*_SGI1_) was transformed into TG1 cells containing R16a or pCU999. Four transformant colonies were grown overnight (ON) in 2 ml LB+Cm+Km (selection for both plasmids) and 40 μl of the 10^5^ × diluted cultures (ca. 500–1000 cells) were transferred into 2 ml LB+Cm (selection only for pMSZ1012) and grown ON. This passage step representing ca. 20 generations was repeated five times. Cultures from each passage were titered on LB+Cm and LB+Km plates. The frequency of Cm^R^ and Km^R^ colonies in the fifth passage was tested also by replica plating and the rate of plasmid loss was calculated accordingly.

### Plasmid constructions

Relevant features of plasmids are listed in Supplementary Table S3. The methodology of plasmid constructions is described in detail in Text S1.

### Alignments and phylogenetic tree constuction

For construction of the phylogenetic tree of IncN RepA proteins, the selected RepA sequences were aligned by ClustalW algorithm and the tree was generated by MEGA X software package (39) using the Maximum Likelihood method with the default settings. The tree was drawn to scale with branch lengths measured by the number of substitutions per site. Bootstrapping was repeated 2000 times. For the alignment of IncN2/N3 *oriV* sequences, the Multalin software (40) was applied.

### Construction of strains expressing RepA_SGI1_ or harbouring *att*P site deriving from circularized SGI1

For chromosomal integration of *repA*_SGI1_ and *attP*_SGI1_, the pLOFKm (41) derivative plasmids pMSZ1014 and pMSZ1041 were introduced into *E. coli* strain S17–1 λ*pir*, respectively, which allows the replication and transfer of R6K-based plasmids carrying the *oriT* of RK2. One colony of each transformation was used as donor strain in a standard mating (23) with TG1Nal recipient. The miniTn*10* chromosomal integrants containing the *repA* or *attP* site of SGI1 were selected on LB+Nal+Km plates at 37°C ON. Km^R^Nal^R^ transconjugants were streaked onto LB+Nal+Km plates and tested for Ap^S^ phenotype indicating the loss of plasmid backbone of pMSZ1014 or pMSZ1041 (conservative transposition of the miniTn*10* unit). Single-copy integrations of miniTn*10*::*repA*_SGI1_-Km^R^ or miniTn*10*::*attP*_SGI1_-Km^R^ were confirmed by Southern hybridization. Total DNA was extracted from three parallel colonies of both transconjugants and ∼400 ng DNA was digested with multiple restriction enzyme combinations. DNA blots using Hybond-N nylon membrane (Amersham) and labelled DNA probes were made using the DIG DNA Labelling and Detection Kit (Boehringer Mannheim) according to the manufacturer's instructions. DIG-labelled probes were amplified with primers S003_BXhrev-pxisseqrev for miniTn*10*::*repA*_SGI1_-Km^R^ insertion and LJ3-RJ5 for miniTn*10*::*attP*_SGI1_-Km^R^ insertion.

### Mobility shift assay

#### Purification of RepA_SGI1_

Overnight culture of *E. coli* strain Tuner (DE3) (Novagen) transformed with pMSZ1066 (RepA-producer plasmid containing P_T7_::*repA*_SGI1_) was diluted 100 × in 25 ml fresh LB+Ap medium and grown to OD600 of 0.5 at 37°C. The culture was induced with 0.2 mM IPTG at 30°C for 5 h under vigorous shaking. Bacteria were harvested by centrifugation and resuspended in 1 ml buffer (50 mM Tris pH 8.1, 300 mM NaCl, 0.01% Triton X-100) supplemented with 60 μg/ml lysozyme and 30 μl of Complete protease inhibitor cocktail (Roche). Cells were frozen at –70°C, then thawed on ice and sonicated at 50% activity for 4 × 10 s. The lysate was centrifuged at 16 000 × g for 30 min at 4°C and RepA_SGI1_ was purified from the supernatant (cleared lysate) using the Dynabeads^®^ His-Tag Isolation & Pulldown Kit (Novex life Technologies) designed for the isolation of histidine-tagged protein by magnetic separation. The purified protein (final concentration of ca. 600 ng/μl) was kept on ice until use.

#### Labelling of oriV fragments

The high copy pBluescript-derivative plasmids, pMSZ1095 and pMSZ1098 were digested with *Eco*RI-*Bam*HI, while pMSZ1120 was digested with XhoI−SacI to isolate the 209 bp, the 124 bp and the 83 bp DNA fragments containing the complete or partial *oriV* sequences, respectively. 100 ng DNA from each fragment was DIG-labelled by terminal transferase (TdT) using the second Generation DIG Gel Shift Kit (Roche) according to the manufacturer's protocol. The labelling reactions were verified by dot-blotting to ensure that they produced the expected amount of labeled DNAs.

#### EMSA assays

Binding reactions were performed in binding buffer (20 mM Tris pH 8.1, 60 mM NaCl, 40 mM KCl, 0.1 mM EDTA, 1 mM Mg-acetate, 2.5 mM ATP, 1 mM DTT, 5% glycerol), containing1 μg poly [d(I-C)], 0.1 μg poly l-lysine, 0.1 ng labelled DNA and different amounts of purified RepA protein (0, 0.3, 0.6, 1.2, 2.4, 3.6, 6.0 μg) in a final volume of 20 μl. For the binding specificity test, 0.1 ng labeled and 250-fold unlabeled competitor DNA fragments were mixed and 1.2 μg protein was added to the binding reaction. Binding reactions were kept on ice for 15 min and 8 μl samples were loaded onto a 5% non-denaturing polyacrylamide gel. Gel electrophoresis was performed in TBE buffer at 8 V/cm and 4°C. Protein−DNA complexes were electro-transferred and crosslinked to a Hybond-N+ membrane (Amersham) using LKB 2117 electrophoresis unit and Amersham ultraviolet crosslinker, respectively. The DIG-label was detected by chemiluminescence and film exposure using alkaline phosphatase-conjugated anti-DIG antibody and CDP-Star according to the protocol of DIG Gel Shift second generation Kit (Roche).

### RT-qPCR assays for relative quantification of *attB*, *attP* and *repA*

Real-time quantitative PCR assays were used to measure the amount of excised circular SGI1 (*attP*), and the unoccupied integration site (*attB*) per cell in TG1Nal::SGI1-C strain with or without R55^ΔTn^*^6187^*. The amount of *attB* and *attP* were normalized to the amount of single-copy chromosomal gene (*trmE*) in each sample. An *E. coli* strain, TG1Nal::*attP*_SGI1_, containing single chromosomal copies of *att*B and *attP*, was constructed to calibrate the RT-qPCR assay. The LJ3-RJ5, attsgifor2-attsgirev2 and attsgifor2-attsgirev3 primer pairs were used to amplify the 251 bp *attP*, the 257 bp *attB* and the 207 bp *trmE* specific fragments, respectively. The RT-qPCR reactions were performed in a final volume of 10 μl using a LightCycler®96 detection system (Roche). Each reaction mixture contained 1 × qPCRBIO SyGreen Lo-ROX master mix (PCR Biosystems), 400 nM of forward and reverse primers and 1 μl *E. coli* cultures (OD600 = 0.5) as templates. The PCR conditions were as: initial denaturation at 95°C for 3 min; 40 cycles of 95°C for 15 s and 60°C for 30 s. Specificity was confirmed by determining the melting curves and agarose electrophoresis of the final PCR products. At least three reactions were performed for each sample. The relative amount of the amplified *attP* and *attB* sequences was calculated based on the Ct deviation of the experimental sample compared to the control sample (TG1Nal::*attP*_SGI1_) and was expressed in comparison to the calibrator (*trmE*) sequence. Consequently, the ratio of *attP* and *trmE* measured the copy number of excised SGI1 and the ratio of *attB* and *trmE* measured the excision of the island.

The copy number of pMSZ1016 was measured as the ratio of *repA* and *trm*E. The primers SGI_repfor-SGI_reprev were used to amplify the 239 bp *repA* fragment. The qPCR reactions were performed as described above. The relative amount of the amplified *repA* sequences was calculated based on the Ct deviation of an experimental sample compared with the control sample (TG1Nal containing pMSZ1016 and pGMY6 grown without IPTG induction) and was expressed in comparison to the calibrator (*trm*E) sequence.

### SGI1 stability tests

For monitoring the stability of SGI1 in the ‘*rep* mutant’ strains harbouring an IncC plasmid, R55^ΔTn^*^6187^* was conjugated from *E. coli* TG90 into the TG1Nal recipient strains carrying SGI1-C KO mutants for *oriV*, *repA*, P*_S004_* or *FlhDC*_SGI1_. Conjugation assays were carried out in 4–6 replicates as described (23). Transconjugants were selected on LB+Nal+Cm plates and then replica plated onto LB+Sm+Sp plates to count Sm^S^/Sp^S^ (SGI1-free) segregants.

Single transconjugant colonies selected on LB+Nal+Cm+Sm+Sp containing both R55 and SGI1 were grown to a stationary phase (ca. 10^9^ CFU/ml) in LB medium supplemented with appropriate antibiotics. Subsequently, 40 μl cultures from the 10^3^ × dilution were transferred into 2 ml fresh medium and grown again to stationary phase without antibiotic selection. Total cell count was determined on LB+Nal plates in four replicates, while the proportion of cells retaining R55 and/or SGI1 was determined by replica plating onto LB+Nal+Cm and LB+Nal+Sm+Sp and LB+Nal+Cm+Sm+Sp plates. The percentage of bacteria harbouring R55, SGI1 or both was calculated as Cm^R^Sm^S^Sp^S^/Nal^R^, Cm^S^Sm^R^Sp^R^/Nal^R^ and Cm^R^Sm^R^Sp^R^/Nal^R^ colonies. The Cm^S^Sm^S^Sp^S^/Nal^R^ ratio showed the subpopulation of cells that lost both SGI1 and R55.

## RESULTS

### SGI1 encodes for a functional RepA protein related to the replication initiators of IncN plasmids

ORF *S003* of SGI1 has been annotated as a *repA* gene (31), but the role of its putative protein product has never been demonstrated. The *repA* transcript was previously reported to be one of the less abundant mRNAs synthesized from a chromosomally integrated island (24) and it was not clear whether this gene is functional at all. Pfam search indicated that the putative RepA protein of SGI1 belongs to the RepA_C-domain-containing proteins and further comparative analysis suggested that it is related to the replication initiator proteins of the broad host range plasmids of IncN family (42) (Supplementary Figure S1). BLASTp search using the amino acid sequence of RepA_SGI1_ showed 10–60% similarities to IncN RepA proteins, depending on the subgroup of IncN family. RepA_SGI1_ significantly differed (<10% identity) from the RepA proteins of IncN1 plasmids (43), and appeared much more similar (50–60% identity) to those of IncN2 group (42,41) and pN-*Cit* classified as IncN3 (44). The phylogenetic reconstruction based on the RepA proteins did not support the separation of IncN2 and IncN3 groups, while IncN1 was clearly distinct from them and possibly forms a different family. Although RepA_SGI1_ was located on a separate branch of the tree along with several RepA proteins of unclassified plasmids, this cluster appeared more closely related to the IncN2 and IncN3 than to the IncN1 group.

To examine the functionality of the putative IncN-related replicon, the *repA* gene of SGI1 was cloned into the p15A-based expression vector, pJKI391, and placed under the control of P_tac_ promoter. The resulting RepA_SGI1_-producer plasmid pGMY9 was transformed into the *E. coli* strain TG1Nal::SGI1-C and the transformants were selected for SGI1 (Sm^R^Sp^R^) and pGMY9 (Km^R^). No transformants were obtained (<1 CFU/0.1 μg pGMY9) even though the expression of RepA_SGI1_ from pGMY9 was achieved by leakage of P*_tac_* (15), while the non-expressing control, pJKI391, resulted in 5 × 10^4^ CFU/0.1 μg DNA under the same conditions. We hypothesized that expression of an active RepA protein in a strain harbouring the cognate *oriV* on its chromosome is severely harmful or even lethal for the host, which might explain the negative result. If this is true, the transformants should be viable when the same *oriV* is located extrachromosomally. SGI1 was known to be excised in the presence of IncC plasmids (14), i.e. they ensure the extrachromosomal location of SGI1 including its *oriV*. Therefore, the transformation of pGMY9 was repeated into the same host but containing the IncC plasmid R55^ΔTn^*^6187^*, and the transformants were selected for SGI1 (Sm^R^/Sp^R^), R55 (Cm^R^) and pGMY9 (Km^R^). As expected, triple-resistant transformants were obtained and their rate was ca. 5 × 10^3^ CFU/0.1 μg DNA.

**Figure 2.**
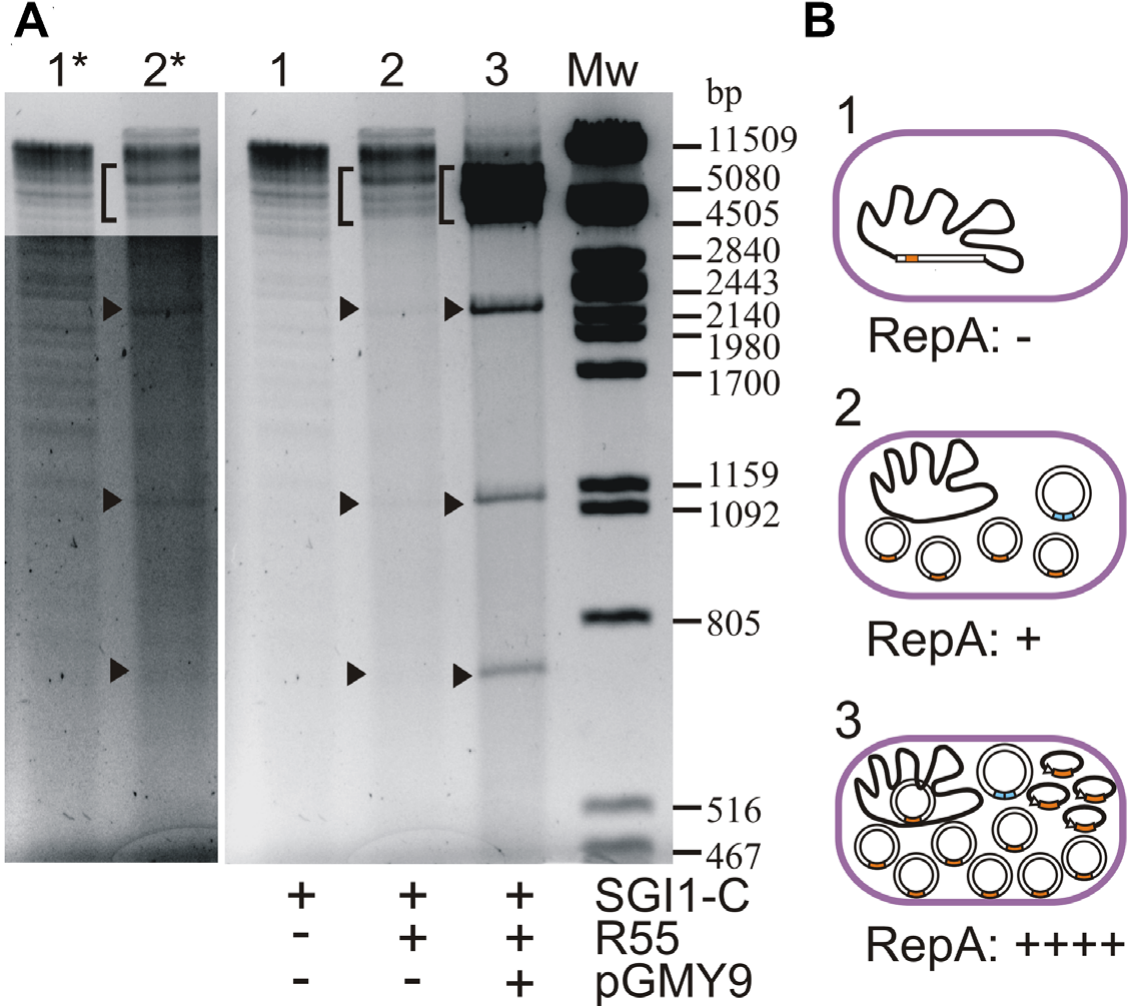
Replication of SGI1-C in the presence of R55^ΔTn^*^6187^* with or without excess RepA. (**A**) Plasmid DNA was extracted from TG1Nal::SGI1-C (lane 1), TG1Nal::SGI1-C/R55^ΔTn^*^6187^*(lane 2) TG1Nal::SGI1-C/R55^ΔTn^*^6187^*+pGMY9 (lane 3) and digested with *Pst*I. Black arrowheads point to the characteristic 2.26, 1.16 and 0.73 kb fragments deriving from the circular SGI1-C. The bracket shows the additional SGI1 fragments (7.75, 7.05, 5.27, 4.49, 4.25 kb) with or without the 6.3 kb linearized RepA producer plasmid, pGMY9. For comparison, lanes 1* and 2* shows the original lanes 1 and 2 with high contrast to make the fragments of the very low copy plasmid-like SGI1 visible. (**B**) Schematic representation of the cells in the three populations of panel A. SGI1 is shown as a bar integrated into the chromosome or as free circular plasmids. *RepA* gene is indicated as an orange box. The helper is shown as a single plasmid with the *acaCD* activator gene (blue bars) required for triggering RepA expression from SGI1, while pGMY9 expressing RepA_SGI1_ from the P_tac_ promoter (open arrowhead) is drawn as an ellipse.

To prove the replication of SGI1-C, plasmid DNA was isolated from transformant colonies and analyzed on agarose gel. SGI1-specific bands were not observed without R55^ΔTn^*^6187^* and pGMY9, but in the presence of both plasmids, strong SGI1-specific fragments appeared along with the band characteristic for pGMY9 (Figure 2A, lanes 1, 3). This confirmed that the *repA* gene of SGI1 encodes for an active RepA protein, which promotes the replication of the excised circular SGI1 and also proved that SGI1 has a functional *oriV*. On the other hand, in the presence of R55^ΔTn^*^6187^* without pGMY9 (lack of excess RepA), the excised SGI1 could not reach such a high copy number observed with pGMY9, however, the weak SGI1-derived bands, especially the characteristic 1.16 and 2.26 kb fragments, were also visible in the plasmid preparation from TG1Nal::SGI1-C/R55^ΔTn^*^6187^* strain (Figure 2, lanes 2 and 2*). This result provided the first hint that SGI1 transiently replicates when coexists with the mobilization helper. Furthermore, the fact that the quantity of extrachromosomal SGI1 was increased by excess RepA_SGI1_ produced from pGMY9 indicated that the copy number depends on the amount of RepA (Figure 2B).

### Determination of the copy number of SGI1

**Figure 3.**
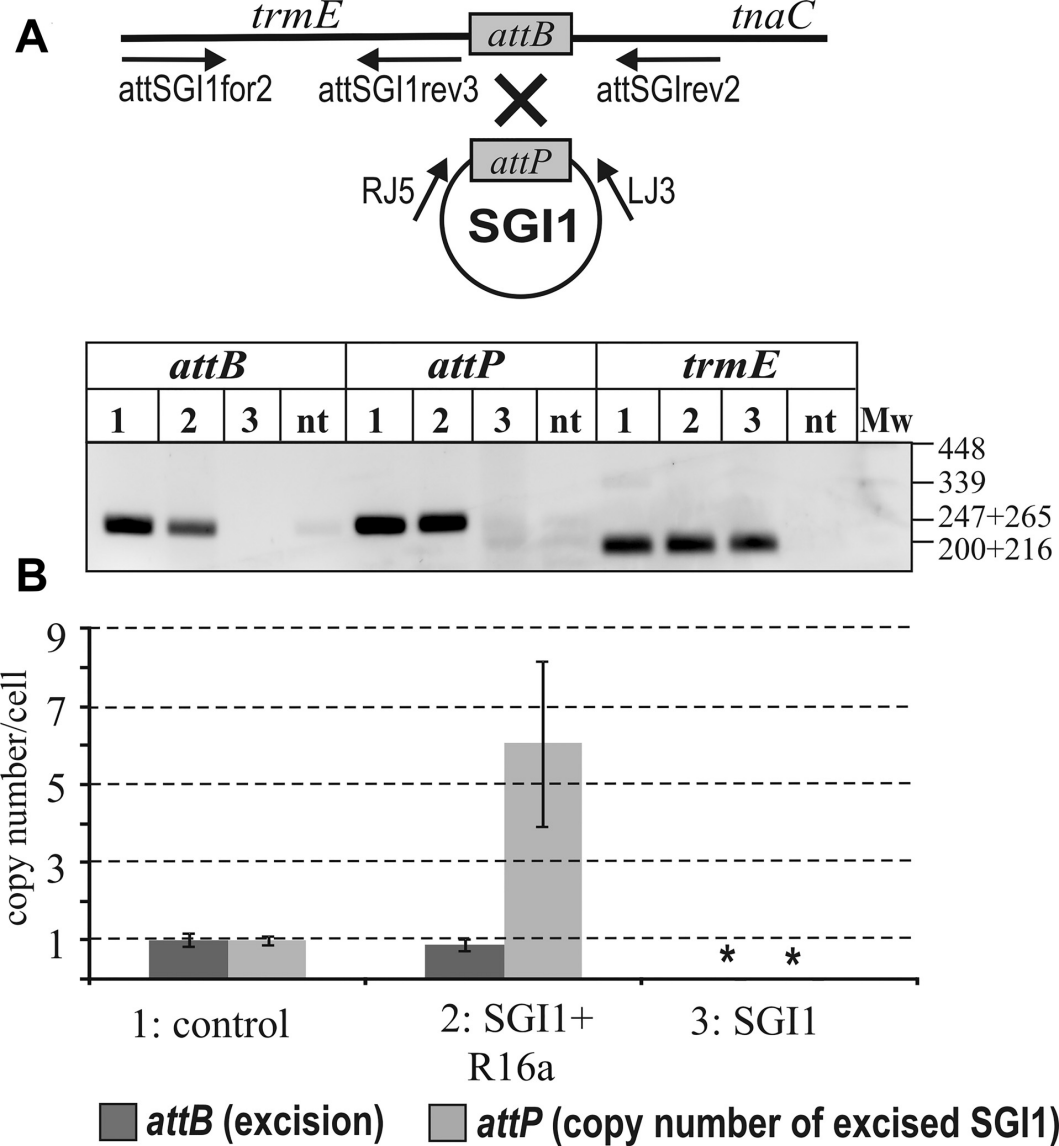
Determination of the copy number of SGI1 by RT-qPCR. (**A**) Schematic representation of the excised SGI1 and the chromosome. The position and direction of primers used are indicated. For measuring *attP* (251 bp), *attB* (257 bp) and the single-copy gene *trmE* (207 bp), the LJ3-RJ5, attsgifor2-attsgirev2 and attsgifor2-attsgirev3 primer pairs were amplified. (**B**) The graph shows the copy number per cell of *attB* and *attP* in the control strain TG1Nal::*attP*_SGI1_ (1), TG1Nal::SGI1-C/R55^ΔTn^*^6187^* (2) and TG1Nal::SGI1-C strain (3). The final PCR products are shown above the graph. nt: non-template control. Asterisks indicate that excision and free circular SGI1 were not detectable in absence of helper plasmid.

As observed in the presence of a helper plasmid, the extrachromosomal form of SGI1 was barely detectable on agarose gel, suggesting that the circular SGI1 is present in the cells as a low copy plasmid (Figure 2). To determine the copy number of the excised plasmid-like SGI1, a real-time quantitative PCR-based analysis was performed. In the RT-qPCR assay, the amounts of unoccupied *attB* sites and the *attP* sequences representing the excised circular form of SGI1 (Figure 3A) were measured in the presence or absence of the IncC helper plasmid and were normalized to the copy number of chromosomal DNA, determined by amplifying the 3′ end of the single-copy gene *trmE*. The same assay was also performed with an SGI1-free control *E. coli* strain, TG1Nal::*attP*_SGI1_, containing single chromosomal copies of *attB* and *attP*. As expected, in absence of the helper plasmid, neither *attB* nor *attP* were detectable in cells containing SGI1-C. This supported the previous observation that Int alone inefficiently catalyze SGI1 excision which can be detected only by nested PCR (19). In contrast, when the R55^ΔTn^*^6187^* helper plasmid was present in the cells, SGI1 was excised in more than 90% of the population. Moreover, the amount of *attP* per cell was 4–8 times higher in this population than in the control strain TG1Nal::*attP*_SGI1_, referring to a significantly increased copy number of the free circular SGI1 form (Figure 3B). These findings clearly indicated a helper-dependent transient replication of the excised island, maintaining an average of 6.6±1.9 extrachromosomal SGI1 copies per cell.

### Identification of *oriV* sequence of SGI1

**Figure 4.**
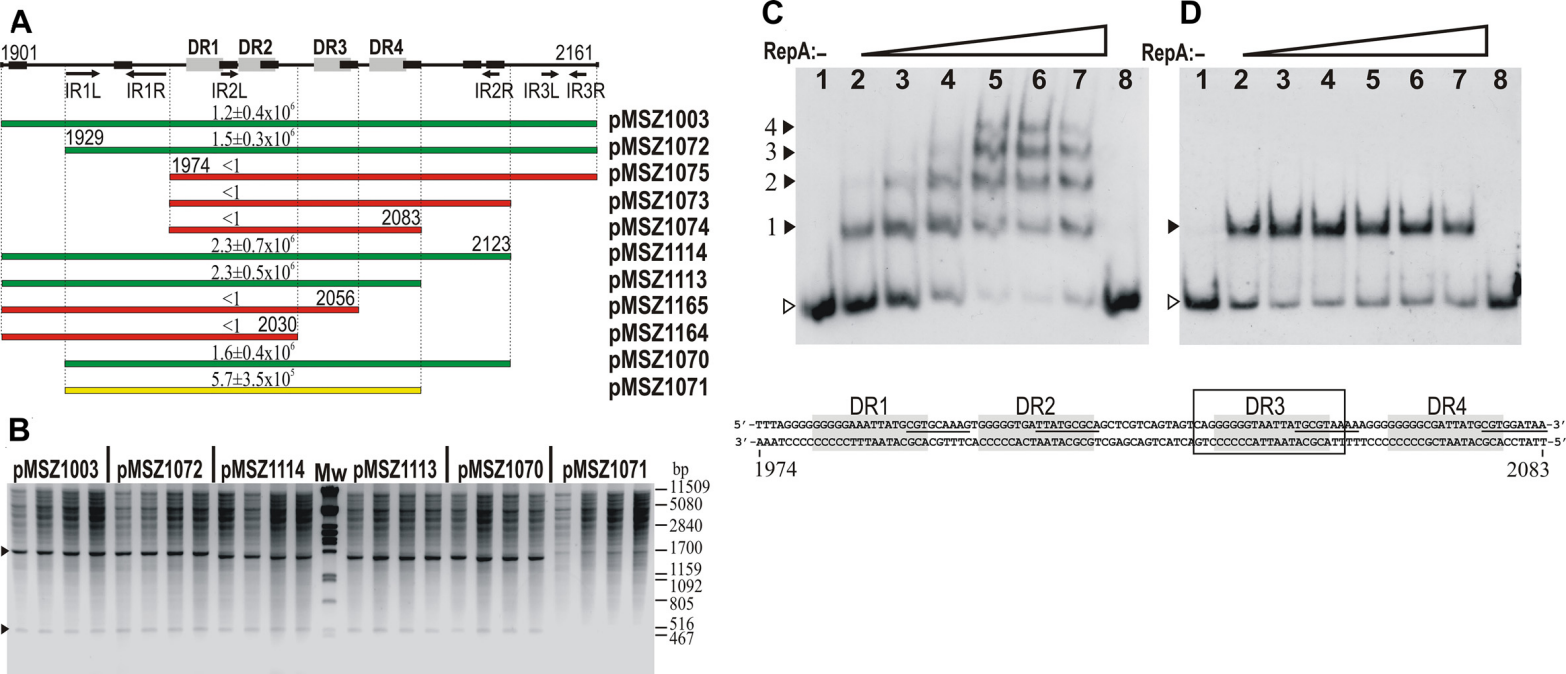
Deletion and EMSA analysis of *oriV*_SGI1_. (**A**) Schematic map of *oriV* region cloned in pMSZ1003 and its shortened derivatives. Coordinates represent the distance in bp from the first position of SGI1 DRL. Grey boxes: DR-s of the iteron-like element of *oriV*, black boxes: putative DnaA binding sites. Left and right parts of inverted repeat motifs (IRL and IRR, respectively) are indicated by arrows. Coloured bars below the map represent the shortened *oriV* fragments. Green: replication/copy number in TG1Nal::*repA*_SGI1_ is similar to that of pMSZ1003, red: non-replicable, yellow: replicable, but with lower copy number compared to pMSZ1003. Transformation efficiencies shown above the bars are given in CFU/100 ng plasmid DNA. (**B**) DNA of replicable plasmids containing shortened *oriV* segments. Plasmid DNA isolated from four parallel Cm^R^ TG1Nal::*repA*_SGI1_ transformants was digested with *Eco*RI-*Pvu*II. Arrowheads point to the resulting fragments of the respective plasmids. (**C**) EMSA for detection of DNA-RepA complex formation using the 124 bp *oriV*_SGI1_ fragment containing the four DRs (gray boxes in the sequence below the images). The putative DnaA boxes are underlined. (**D**) EMSA for RepA binding to the fragment containing the single DR3 repeat (boxed in the sequence below the images). The amount of RepA protein was increased from 0 to 6 μg (lanes 1–7: 0, 0.3, 0.6, 1.2, 2.4, 3.6 and 6.0 μg). In the binding specificity tests (lanes 8), 250× unlabeled DNA fragment was added to the binding reaction shown in lanes 4. Filled arrowheads point to the shifted complexes, open arrowheads indicate the unbound probe.

During the previous analysis of the noncoding region between *xis* and *repA*, imperfect direct repeat (DR) motifs were found, which proved not to be involved in the regulation of *xis* gene (14), but resembled the iteron-like repeats of *oriV* regions of many plasmids (45,46). Based on the homologies with IncN2/N3 plasmids, the putative *oriV* was predicted downstream of *repA*_SGI1_. BLASTn alignment of downstream sequences of *repA* in the related plasmids and SGI1 revealed a ca. 70% conserved 185-bp noncoding region beginning 50 bp downstream of the STOP codon of *repA*_SGI1_ (Supplementary Figure S2). This region includes six putative DnaA binding sites, a 15-bp inverted repeat (IR) motif and the previously recognized iteron-like segment with four 16-bp DR motifs (Figure 4A). These DRs differ at several positions and can be characterized by the GGGGGHRATTATGCGY consensus.

To examine the functionality of the predicted *oriV*, the 1901–2161 bp region located downstream of *repA*_SGI1_, including the conserved segment and additional two IR motifs and two putative DnaA-boxes, was cloned into a conditionally replicating R6K-based Cm^R^ vector pSG76-CS. The resulting plasmid, pMSZ1003, and the parental pSG76-CS vector were introduced into the *E. coli* strain TG1Nal::*repA*_SGI1_, which expresses the RepA_SGI1_ protein from a chromosomally integrated P_tac_::*repA* cassette but does not support the maintenance of R6K-based replicons. While no Cm^R^ transformants were obtained with pSG76-CS, transformation with pMSZ1003 resulted in 1.2±0.4 × 10^6^ CFU/0.1 μg DNA. Restriction mapping of plasmid DNA from several transformant colonies confirmed the presence of pMSZ1003 (Supplementary Figure S3A), indicating that the cloned fragment contains the functional *oriV*_SGI1_.

As a next step, the P_tac_::*repA* cassette was inserted into pMSZ1003 and the resulting pMSZ1012 was introduced into the *polA* mutant *E. coli* strain and its isogenic wild type counterpart (ME6266 and ME7772, respectively). The transformation resulted in ca. 10^4^ Cm^R^ CFU/0.1 μg DNA in both strains. The plasmid DNA isolated from several colonies also indicated that pMSZ1012 replicates in a PolA-independent manner (Supplementary Figure S3B). The SGI1-derived minimal replicon was fully replicable in *E. coli* strain TG1 containing the Km^R^ pCU999, a derivative of the IncN1 plasmid pCU1, supported by the analysis of plasmid DNA isolated from TG1/pCU999+pMSZ1012 cells (Supplementary Figure S3C). In addition, a compatibility test was performed with these transformants. No pCU999 loss (<0.5±0.3%) was observed during 100 generations in the presence of pMSZ1012 without a selection for pCU999, confirming their compatibility. A similar assay was carried out with the IncC plasmid R16a, which also proved to be stable together with the minimal SGI1 replicon (loss of R16a was not detected, <0.7±0.2%).

In order to further specify the minimal *oriV*_SGI1_, a deletion analysis was performed. Maintenance of pMSZ1003-like plasmids carrying shortened *oriV* regions was tested in the RepA expressing strain TG1Nal::*repA*_SGI1_ as described above. Transformation of plasmids pMSZ1073, pMSZ1074, pMSZ1075 carrying the four DRs with different lengths of the 3′ flanking region but lacking the IR1 motif resulted in no Cm^R^ transformants, indicating that these constructs are not able to replicate even in the presence of RepA_SGI1_ (Figure 4A). Similarly, the plasmids pMSZ1165 and pMSZ1164, lacking one or two copies of the DR motifs, could not replicate. In contrast, all the other clones resulted in viable transformants. The shortest replicable plasmid was pMSZ1071 possessing a 155-bp sequence extending from 1929 to 2083 bp of SGI1. Nevertheless, this construct resulted in less and smaller colonies than the others. Moreover, the pMSZ1071 transformants contained less plasmid DNA, referring to its lower copy number (Figure 4B). pMSZ1070, containing the *oriV* segment from IR1L to IR2R and pMSZ1113, lacking IR2R but containing an additional 28 bp flanking the IR1L at the 5′ end, replicated similarly to any other constructs harbouring longer *oriV* segment. Interestingly, pMSZ1071 containing the common region of the fully replicable pMSZ1070 and pMSZ1113 was probably less stable (gave smaller colonies) and maintained at a lower copy number. Altogether, we showed that the 1929–1973 bp AT-rich region with the 15 bp imperfect inverted repeat (IR1R has 3 bp insertion compared to IR1L) and the well-conserved 1974–2083 bp core region containing four DRs and the five potential DnaA binding sites are essential for SGI1 replication. The 2084–2123 bp region, which is highly conserved in IncN2/N3 plasmids (Supplementary Figure S2), and the 1901–1928 bp sequence may contain auxiliary sequences (possibly the further three DnaA sites) that are not essential but can facilitate the replication.

### Detection of RepA-binding to *oriV*

The sequence-specific interaction of RepA_SGI1_ with the *oriV* region was investigated by EMSA using the purified His-tagged RepA_SGI1_ protein (Supplementary Figure S4) and different fragments of *oriV*_SGI1_. First, the fully replicable fragment of SGI1 containing the most conserved part of *oriV*_SGI1_ (1929–2123 bp cloned in pMSZ1070, Figure 4) was end-labelled and used to optimize the binding conditions. When using a higher amount (≥2.4 μg) of RepA protein, four slower migrating complexes were detected (not shown), indicating that four binding sites occur on this fragment. We assumed that the four binding sites correspond to the four 16-bp DRs located in the central region of *oriV*, thus in the second binding assay, the labelled DNA probe was shortened to the four DRs of *oriV* (1974–2083 bp). As expected, increased amount (0.3–6 μg) of RepA protein also resulted in four retarded bands indicating the consecutive filling of the four binding sites (Figure 4C). To test whether the RepA binds to a single DR sequence, the 2035–2055 bp SGI region containing the DR3 was used in EMSA. The shifted band already appeared when using 0.3 μg RepA and no further complexes were detected with higher amounts of the protein (Figure 4D).

### The role of FlhDC-family activators in SGI1 replication

**Figure 5.**
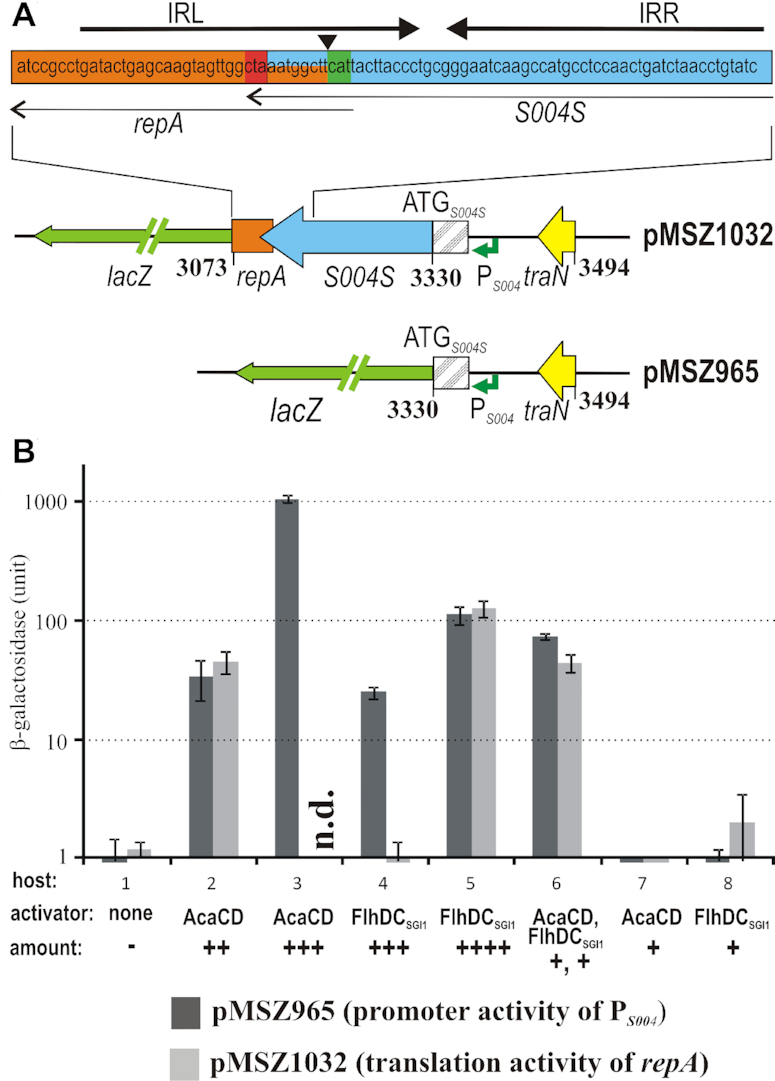
β-galactosidase assay for measuring the expression of LacZ-RepA fusion protein and the promoter activity of P*_S004_* in host strains expressing the FlhDC-family activators from different sources. The schematic map of the relevant part of test plasmids pMSZ1032 and pMSZ965 are shown. Plasmid pMSZ1032 contains a translational *repA*-*lac*Z fusion (codon 14 of *repA* is followed by codon 8 of *lacZ*) under the control of P*_S004_*, thus the amount of β-galactosidase produced under the different conditions refers to the translation level of RepA. The *lacZ* gene is indicated by a green arrow, the untranslated part of ORF *S004* is hatched. The plasmids carry the 3′ part of *traN* only. Other symbols and colour coding are as in Figure 1. The coordinates refer to the start- and endpoints of the respective SGI1 regions and the position of START codon of *S004S*. The IR sequence motif that can form a putative stem-loop structure on the mRNA is shown above the plasmid map. STOP and START codons of the overlapping *repA* and *S004S* are indicated as red and green, respectively. Position of *lacZ* fusion in the test plasmid, which was proved previously to be negative in β-galactosidase expression (33) is indicated by the filled arrowhead. In pMSZ965, *lacZ* is fused to the start codon of ORF *S004S* under the control of P*_S004_* to measure the activity of P*_S004_*. The graph shows the results of the β-galactosidase assay, the data were obtained from four parallel pMSZ965 and pMSZ1032 transformants. Host strains were: 1, TG1Nal (no activator); 2: TG1Nal/pJKI828 (*acaCD* in the original operon); 3, TG1Nal/pJKI888 (P_tac_::*acaCD*, non-induced); 4, TG1Nal/pGMY6 (P_tac_::*flhDC*_SGI1_, non-induced), 5, TG1Nal/pGMY6 (P_tac_::*flhDC*_SGI1_, 0.05 mM IPTG-induced), 6, TG1Nal::SGI1-C^Δrep_region^/R55^ΔTn^*^6187^* (*acaCD+flhDC*_SGI1_), 7, TG1Nal::SGI1-C^Δrep_region^*^+^^Δ^^flhDC^*/R55^ΔTn^*^6187^* (*acaCD*), 8, TG1Nal::SGI1-C^Δrep_region^/R55^ΔTn^*^6187+^^Δ^^acaCD^* (*flhDC*_SGI1_). n.d. – not determined due to the lack of viable transformants.

The results described above clearly suggest that SGI1 is capable for plasmid-like replication, which occurs only in the presence of the helper plasmid. Thus, we assumed that IncC plasmids can induce SGI1 replication. The last four codons of ORF *S004* overlap with *repA* and this region is located inside of a long IR motif that can form a potential stem-loop structure on the mRNA transcript (Figure 5A). Similar genetic constitution often occurs in different plasmid families, where a leader protein contributes to the control of *repA* expression (47). The translation of leader peptides is required for or enhances the translation of the downstream reading frames (48). This analogy and our previous observation that expression of ORF *S004* is activated by AcaCD and FlhDC_SGI1_ (33) suggest that *repA* is expressed as a part of the *S004-repA* operon under the control of the AcaCD-responsive promoter P*_S004_*. Since the expression from a test plasmid carrying the *lacZ* gene fused to the START codon of *repA* was not detectable even in the presence of excess AcaCD (33), the possibility of AcaCD/FlhDC_SGI1_-controlled *repA* expression was re-examined here using newly designed plasmid constructs (Figure 5A). For this, two methods were applied: (i) the translation of *repA* gene and *S004* was monitored by β-galactosidase assays in hosts producing one or both activators and (ii) the replication ability of the SGI1-derived minimal replicon was tested under the same conditions.

For the first approach, two β-gal tester plasmids were constructed where the *lacZ* expression was controlled by the AcaCD-responsive promoter P*_S004_*, the putative promoter of the *S004-repA* operon. Plasmid pMSZ1032 contained a translational *repA*-*lacZ* fusion (the codon 14 of *repA*_SGI1_ was fused in frame to the codon 8 of *lac*Z), retaining the long IR motif. Therefore, the β-galactosidase activity measured with pMSZ1032 referred to the translation level of RepA_SGI1_. In the other tester plasmid, pMSZ965, the START codon of *lacZ* was placed at the ATG of ORF *S004S*, being the valid START site of the protein translated from ORF *S004* (33). Thus, it measured the translation level of S004 referring to the activity of P*_S004_* (Figure 5A). The β-galactosidase assays were carried out with both constructs in host strains, which provided the AcaCD and/or FlhDC_SGI1_ activators from expression plasmids or, as a more realistic model of the ‘native’ situation, from the helper plasmid R55^ΔTn^*^6187^* and SGI1. In the latter case, FlhDC_SGI1_ was supplied by a deletion mutant SGI1-C lacking P*_xis_* and the whole rep region (SGI1-C^Δrep_region^). This prevented both the excision and additional RepA expression but did not affect the FlhDC_SGI1_-production of the island. The β-galactosidase activity of pMSZ965 was undetectable in the absence of both activators, while it was measurable either in the presence of any p15A-based producer plasmids providing the activators in excess or if both activators were supplied from their original sources. In contrast, transcription from P*_S004_* was undetectable in hosts with Δ*acaCD* or Δ*flhDC*_SGI1_ background, indicating that none of the activators could induce P*_S004_* alone at a concentration supplied by the helper plasmid or SGI1 (Figure 5B). Translation measurements using pMSZ1032 correlated well with the transcriptional activities of pMSZ965 in most cases, indicating that RepA production is under the control of P*_S004_* and its activators. One exception was the non-induced FlhDC_SGI1_-producer host, in which the promoter activity was well detectable (24.9±2.8 units of β-gal) while the translation activity was lacking (≤0.8±0.6 units of β-gal). Interestingly, pMSZ1032 could not be introduced into the TG1Nal/pJKI888 strain expressing AcaCD from P_tac_ even without IPTG-induction, which could be due to the cumulative negative effect of the overexpressed AcaCD and the high level of AcaCD-induced S004S protein (33) on the bacterial growth. Thus, in order to detect the translational activity, a lower level of AcaCD expression was achieved using pJKI828. This plasmid contains the entire operon encoding AcaCD under the feedback control of the repressor Acr1 (22) and previously has been shown to cause much weaker expression than the uninduced pJKI888 producing AcaCD by leaking of P_tac_ (14). Our data supported the previous observations that the efficiency of the two activators is different (33) (compare hosts 3 and 4 in Figure 5B) and the expression level of RepA depends on the amount of activators. Furthermore, both activators were necessary for a detectable RepA expression if they were supplied from the helper plasmid and SGI1 under their original expression control.

As a second approach, the maintenance and copy number of the SGI1-derived minimal replicon were investigated in the activator-producing hosts applied in β-gal assays. The suspected minimal functional rep region of SGI1 (1901–3494 bp including the *oriV* sequence and the *S004*-*repA* operon with the −10 box and the AcaCD-binding site of P*_S004_*) was joined to a Cm^R^ gene (Supplementary Figure S5). Therefore, maintenance of the resulting construct pMSZ1016 was possible only if the expression of RepA was induced by AcaCD and/or FlhDC_SGI1_ through the promoter P*_S004_*. In case of the p15A-based producer plasmids, the impact of different amounts of the activators was examined. In hosts carrying a helper plasmid, R55^ΔTn^*^6187^* was replaced with R16a due to its more convenient resistance. Replication of pMSZ1016 in these hosts was monitored by determining transformant frequencies, RT-qPCR-based copy number measurement and analysis of plasmid DNA prepared from viable transformants (Table 1, Supplementary Figure S5). The results correlated well with the outcome of β-gal assays: transformants were obtained only if either activator was expressed from a p15A-based producer plasmid (AcaCD from pJKI828 or pJKI888 and FlhDC_SGI1_ from IPTG-induced pGMY6) or both were expressed from R16a and the chromosomally integrated SGI1-C^Δrep_region^ (Table 1). As expected, pMSZ1016 proved not to be replicable (i.e. no transformant colonies were obtained) in hosts with Δ*acaCD* or Δ*flhDC*_SGI1_ background. This supported again the conclusion that both activators are required for SGI1 replication under the quasi-natural situation when they are supplied by the coexisting helper plasmid and SGI1. In this case, pMSZ1016 was maintained in a similar copy number (4–5/cell) measured previously for the wt SGI1 in the presence of R55^ΔTn^*^6187^*, while higher copy numbers were observed if either AcaCD or FlhDC_SGI1_ alone was present in excess. Unlike FlhDC_SGI1_, overexpression of AcaCD proved to be lethal for pMSZ1016 transformants likely due to the same reason as for pMSZ1032 in β-gal assays. Therefore, pJKI828 producing a lower level of AcaCD was used for comparison with the uninduced pJKI888. The frequency of the transformants and their pMSZ1016-content were proportional to the amount of the activators. FlhDC_SGI1_ alone induced the replication less efficiently than AcaCD, as pMSZ1016 could only be maintained by IPTG-induced pGMY6 (Table 1, Supplementary Figure S5). In conclusion, the copy number of SGI1, at least in a certain range, is proportional to the amount of RepA, which depends on the amount of the activators. Furthermore, unlike the excision induction (14), both AcaCD and FlhDC_SGI1_ are required for proper replication of SGI1 under natural conditions.

**Table 1.** Transformability and copy number of the SGI1-derived replicon pMSZ1016 in strains expressing different amounts of the transcriptional activators AcaDC and FlhDC_SGI1_

strain	TG1Nal	TG1Nal/ pJKI828	TG1Nal/ pJKI888 no induction	TG1Nal/ pGMY6 no induction	TG1Nal/ pGMY6+ 50μM IPTG	TG1Nal:: SGI1^Δrep_region^/ wt R16a	TG1Nal:: SGI1^Δrep_region+Δ^*^flhDC^*/ wt R16a	TG1Nal:: SGI1^Δrep_region^/ R16a^Δ^*^a^^c^^a^^CD^*
Activator	None	AcaCD	AcaCD	FlhDC_SGI1_	FlhDC_SGI1_	AcaCD+FlhDC_SGI1_	AcaCD	FlhDC_SGI1_
Amount	−	++	+++	+++	++++	+, +	+	+
Transformants (CFU/0.1μg of pMSZ1016)	<1	9.1±2.1 × 10^2^	2.1±1.4 × 10^4^	<1	8.0±3.2 × 10^4^	2.3±1.5 × 10^3^	<1	<1
Copy number of pMSZ1016	−	2.4±0.5^a^	13.2±1.9	1.0±0.2^b^	31.8±2.5	4.4±1.4	-	-

^a^The copy number measurement was carried out under non-inductive conditions using transformant colonies obtained from plates supplemented with 50 μM IPTG

^b^Transformants obtained with the host containing pJKI828 grew considerably slower compared to that carrying pJKI888.

### Translation of *S004S* is required for RepA synthesis

If S004S protein acts as a classical leader peptide, its translation is required to normal expression of RepA. Consequently, nonsense mutations in ORF *S004S* should decrease or prevent RepA synthesis. To test this hypothesis, the 24th Leu codon of ORF *S004S* was replaced with a UAA (ochre) stop codon in both test plasmids, pMSZ1016 and pMSZ1032 (resulting in plasmids pMSZ1028 and pMSZ1034, respectively). pMSZ1028 was used to detect the replication ability of the SGI1 rep region without S004S protein as described for pMSZ1016, while pMSZ1034 was applied to monitor the expression level of RepA by β-galactosidase assay. Although pMSZ1028 could be maintained in strain TG1Nal::*repA*_SGI1_, in contrast to its parental plasmid pMSZ1016, no pMSZ1028 transformants were obtained within the strain TG1Nal::SGI-C^Δrep_region^/R16a (producing both activators) even with expressing the S004S protein from plasmid pMSZ1040 containing the P_tac_::*S004S* cassette (33). Thus, the nonsense mutation in *S004S* prevented the replication and this deficiency was not rescued by S004S protein overexpressed *in trans*. Similarly, pMSZ1028 did not replicate in any activator-producing strains independently of the expression levels.

The β-galactosidase assay showed very low *lacZ* expression from pMSZ1034, which was near the detection limit (0.8±0.5 units) and it was not increased (1.1±0.4 units) by S004S protein overexpressed from pMSZ1040, indicating that RepA synthesis is very limited in the absence of S004S translation *in cis*. Since β-galactosidase expression from the wt parental plasmid pMSZ1032 was relatively low (44.2±7.1 units) in the host harbouring R55 and SGI1, a more sensitive test was also applied. For this, the AcaCD-inducible P*_S004_* promoter of pMSZ1032 and pMSZ1034 was replaced with the P_tac_ promoter to ensure elevated expression of the *repA*-*lac*Z fusion. The resulting plasmid pMSZ1037 bearing wt *S004S* ORF produced 118.8±4.5 units of β-galactosidase without IPTG induction, and 1612.7±81.8 units after induction with 0.05 mM IPTG. Contrarily, pMSZ1039 containing a stop codon in *S004S* produced only 0.7±0.3 and 2.2±0.6 units under the same conditions, and the mutation was not complemented by S004S protein overexpressed from pMSZ1040. These results confirmed that the translation of S004S *in cis* is essential for RepA synthesis from the *S004-repA* operon even if it is placed under the control of a strong promoter like P_tac_.

### Replication has a key role in the stability of SGI1 in the presence of IncC helpers

Transient replication of the extrachromosomal form of ICEs has been shown to contribute to the stable maintenance of the islands (27). In most of these cases, relaxase-dependent RC-replication occurs, while the SGI1-derived replicon proved to belong to the theta-type IncN2/N3-family. Nevertheless, we supposed that the helper-induced replication of SGI1 has a similar role in SGI1 maintenance when it coexists with the mobilization helper. To test this hypothesis, deletions affecting the replication functions of the island were generated in TG1Nal::SGI1-C strain and these mutants were used to monitor their co-habitation with the helper plasmid. In addition to knocking out the *oriV*_SGI1_, *repA*_SGI1_ and the AcaCD binding site of P*_S004_*, a Δ*flhDC*_SGI1_ mutant was also generated, as FlhDC_SGI1_ appeared to be an important factor in proper replication of pMSZ1016, maintained by the SGI1-derived basic replicon (Table 1).

**Figure 6.**
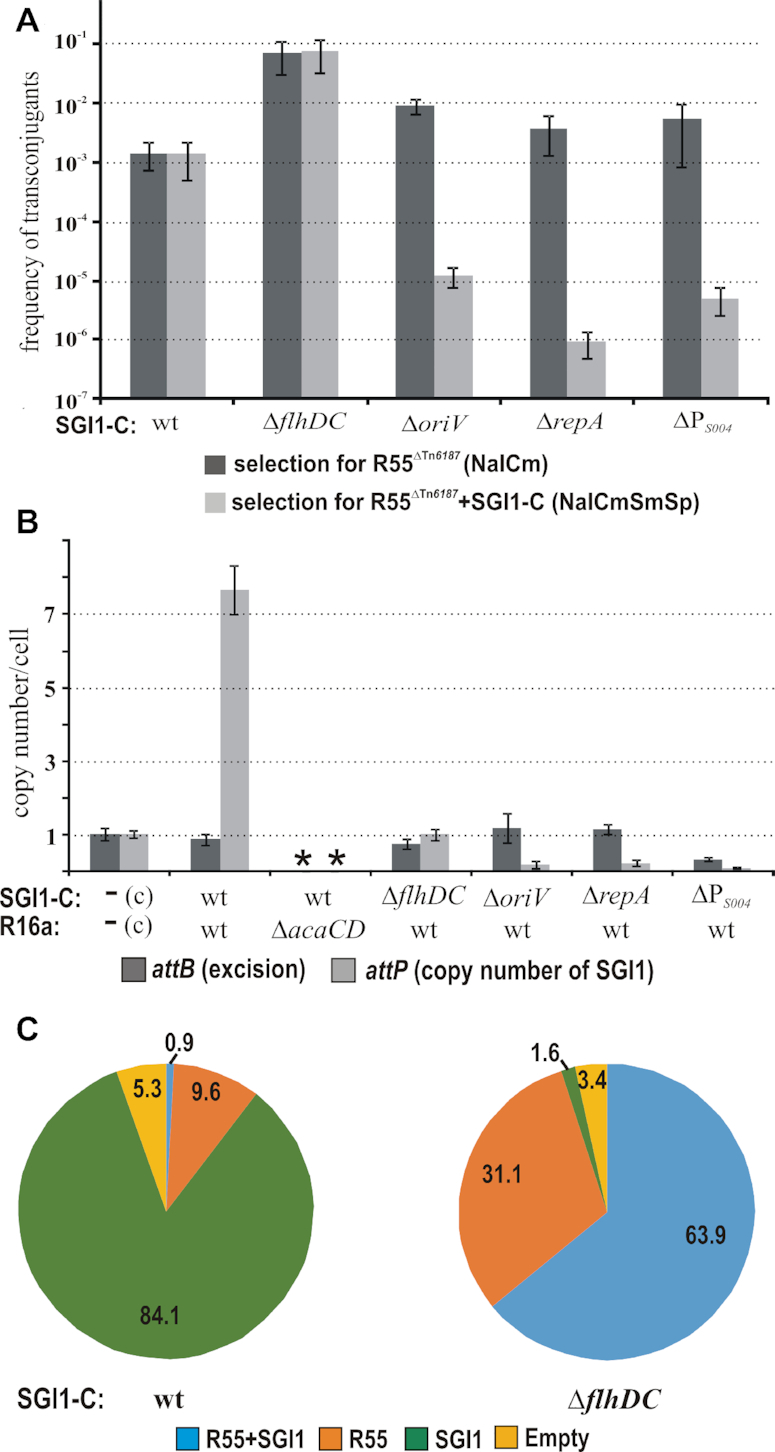
Analysis of the role of SGI1 replication in its stability in the presence of IncC plasmids. (**A**) Retention of replication-deficient SGI1-C mutants besides the IncC plasmid. The graph shows the frequencies of R55^ΔTn^*^6187^* transconjugants obtained from mating that was carried out with or without selection for the wt or KO mutant SGI1-C resident in the recipients. (**B**) Excision and copy number of the extrachromosomal wt or KO mutant SGI1-C in cells harbouring also an IncC plasmid. Data of RT-qPCR were normalized to the control strain (marked with ‘c’ on the chart) containing a single copy of *attB* and *attP*. Asterisks indicate that neither excision nor replication is detectable in absence of AcaCD. (**C**) Segregation of SGI1-C and the IncC helper under nonselective conditions. Diagrams show the distribution of R55^ΔTn^*^6187^* and SGI1-C after 20 generations without selection.

For the first attempt, TG1Nal::SGI1-C strain and its KO mutant derivatives were used as recipients in mating with an IncC plasmid-bearing donor strain TG90/R55^ΔTn^*^6187^* and the frequency of transconjugants was determined under selecting only for the incoming plasmid (LB+Nal+Cm) and for both the plasmid and SGI1 (LB+Nal+Cm+Sm+Sp), respectively. As in any similar assays, the frequency of transconjugants depends not only on the primary rate of transfer but also on the stability of SGI1 and helper plasmid co-habiting in the transconjugant cells. Thus, the transconjugants obtained without selection for SGI1 were also individually tested for the presence of SGI1 by replica plating. The transconjugant frequencies with the recipient bearing wt SGI1-C were similar independently of the selection method (Figure 6A) and the phenotype test showed 11.5±3.7% loss of SGI1 from the transconjugants not selected for the Sm^R^Sp^R^ markers of the island. Similar segregation rate was observed previously in *Salmonella* (14). In contrast, ca. 3 orders of magnitude lower transconjugant frequencies were observed when the recipients carried one of the three replication-deficient SGI1-C mutants (Δ*oriV*, Δ*repA* and ΔP*_S004_*) and the transconjugants were also selected for SGI1 than without selecting for it. The phenotype test of individual R55^ΔTn^*^6187^* transconjugants selected only for the plasmid marker indicated a high rate of SGI1 loss (Δ*oriV*: >99.7±0.1%, Δ*repA*: >99.6±0.1 and ΔP*_S004_*: 96.8±2.0% SGI1-free transconjugants). A characteristic feature of these replica-plated colonies was that only a few small secondary colonies appeared in the footprint of the original transconjugant colonies on the plates selective for both SGI1 and R55 (Supplementary Figure S6). These data indicate that the replication-deficient SGI1 had been lost almost completely from the colonies of the transconjugant cells without a selection for SGI1. These observations confirmed a drastic decrease in the stability of all rep-mutant islands in the presence of the helper plasmid.

### FlhDC_SGI1_ is an important factor in SGI1 replication

The Δ*flhDC*_SGI1_ mutant appeared rather different from the rep mutants in similar tests. The frequency of R55 transconjugants obtained with the recipient containing this mutant was ca. 50 × higher compared to the wt SGI1-containing recipient, independently of the selection (Δ*flhDC*_SGI1_: 7.3±4.1 × 10^−2^ versus 7.5±4.3 × 10^−2^ and wt: 1.5±0.7 × 10^−3^ versus 1.4±0.9 × 10^−3^, Figure 6A). The finding that the SGI1-derived replicon (pMSZ1016) was not maintained in an *flhDC*_SGI1_^−^*acaCD*^+^ background (Table 1) suggested an insufficient replication in the absence of FlhDC_SGI1_. Thus, to determine the copy number of the wt SGI1-C and the Δ*flhDC*_SGI1_mutant in cells containing the IncC plasmid R16a, RT-qPCR assay for *attP* and *attB* was performed, including the three rep mutants. The results confirmed that the wt island is present in 7–8 copies/cell when a wt (*acaCD*^+^) helper is present, while neither excision nor replication was detectable in an *acaCD*^−^ background (Figure 6B). The excision of SGI1 KO-mutants, including Δ*flhDC*_SGI1_, was almost complete in an *acaCD*^+^ background similar to that of wt SGI1-C (>80%, except ΔP*_S004_* mutant showing a somewhat lower, 32±6%, excision rate). The copy number of the excised rep-mutant islands was <0.2 per cell (Figure 6B), indicating their proper excision but the lack of replication. This can explain the frequent SGI1 loss and the low rate of transconjugants retaining SGI1 after the intake of the helper plasmid (Figure 6A). The only exception was the Δ*flhDC*_SGI1_ mutant, whose copy number proved to be around 1/cell. Although the FlhDC_SGI1_ depletion caused a significant reduction in the copy number (from 7–8 to 1/cell), the intact SGI1-C, unlike the SGI1-derived minimal replicon (pMSZ1016), was maintained as a single-copy plasmid.

### Copy-number of SGI1 influences its incompatibility with the helper plasmid

At a first glance, the lower copy number of Δ*flhDC*_SGI1_ mutant cannot explain the 50× higher frequency of R55 transconjugants (Figure 6A) compared to that obtained with the recipient carrying wt SGI1-C. However, the data suggested a more stable co-habitation of the helper plasmid and the island if SGI1 is present in single copy due to the lack of its own activator.

To further analyse this phenomenon, isogenic strains harbouring R55^ΔTn^*^6187^* and wt or Δ*flhDC* mutant SGI1-C were grown for 20 generations without antibiotic selection and the proportion of cells still containing one or both elements was determined. In case of wt SGI1-C, >84% of the cell population carried only SGI1 and <1% retained both partners. In contrast, SGI1-only cells almost disappeared (1.6%) in the case of the Δ*flhDC* mutant and the population was dominated by cells retaining both the plasmid and the island (Figure 6C). These findings suggest that the lack of FlhDC_SGI1_ can contribute to the stability of the co-habitation, which can also explain the higher frequency of R55 transconjugants obtained with SGI1-C^Δ^*^flhDC^*-bearing recipient. Nevertheless, the background of this phenomenon is not yet clarified.

## DISCUSSION

SGI1-family IMEs are known as passengers hijacking the transfer apparatus of IncA and IncC plasmids. Several important characteristics of the relations between the IME and its mobilization helper have recently been uncovered (14,16,22,32,33,49). The present study reports a new aspect of the SGI1-helper crosstalk, which highly contributes to the stability of SGI1 when coexisting with the helper plasmid. We provide evidence for the helper-induced plasmid-like replication of the excised SGI1, which seems indispensable for the maintenance of the island in helper-bearing cells and may also contribute to the efficient displacement of the helper.

The basic replicon identified on SGI1 includes the *S004-repA* (*S003*) operon and the downstream *oriV*. ORF *S003* encodes for a functional replication initiator, which is related to the IncN2/N3 family RepA proteins (Supplementary Figure S1). The cognate *oriV* region identified near the 3′ end of *repA*_SGI1_ includes four iteron-like DRs with a GC-rich GGGGGHRATTATGCGY consensus preceded by an AT-rich region containing the imperfect IR1 motif. This arrangement of *oriV* is characteristic for the iteron-containing replicons (50). Similar conclusions have been reported on the SGI1-derived replicon in a very recent study published after the submission of this work (51). Both *repA* and the *cis* elements of *oriV* are well conserved in the IncN2/N3 group of plasmids having closely related replicon to that of SGI1 (Supplementary Figure S2). The specific protein-DNA interaction between RepA_SGI1_ and the DRs of *oriV* has been confirmed by EMSA, showing that the four DRs serve as binding sites for RepA_SGI1_. The *oriV* region contains putative DnaA-boxes neighbouring or overlapping the DR1–4 motifs. DnaA binding sites are usual components of many prokaryotic replication origins (47), including the IncN1 plasmid pCU1 (52) and the IncN2/N3 plasmids (Supplementary Figure S2). Deletion analysis of *oriV*_SGI1_ confirmed that the conserved DRs and the AT-rich region including IR1 are necessary for replication, however, short regions flanking IR1L and DR4 also have a role in maintaining the maximal copy number driven by *oriV*_SGI1_ in a RepA-expressing strain (Figure 4). The basic replicon of SGI1 proved to be compatible with both the pCU1-derived IncN1 plasmid pCU999 and the IncC plasmid R16a, and its replication is PolA independent. The replicon of the IncN1 plasmid pCU1 was analyzed in detail (52–54), however it is not closely related to and appears to be quite different from that of SGI1 and the IncN2/N3 group. On the other hand, at least to the best of our knowledge, the replication system of IncN2/N3 group including the closest relatives of the SGI1-borne replicon is virtually unexplored. Thus, our study provides the first analysis of an IncN2/N3 replicon, although further work is needed to reveal the molecular mechanisms and control of SGI1 replication.

SGI1 was shown to be maintained and vertically transmitted as an element integrated into the host chromosome (19). Its stability is primarily ensured by the integration activity of Int and the lack of Xis (14), i.e. in the absence of helper plasmids neither the excision of nor free circular SGI1 are detectable (Figure 2). In contrast, we have documented that SGI1 exists as a circular extrachromosomal plasmid-like element in a host also carrying an IncC plasmid and that SGI1 replicates transiently in a helper-induced manner. In this situation, SGI1 is maintained in 4–8 extrachromosomal copies due to the AcaCD-induced expression of RepA as has also been reported recently (51). Therefore, the copy number of SGI1 significantly exceeds that of the IncC helpers, which are known to be very low or even single-copy plasmids (55). The transiently elevated copy number of SGI1 likely provides a variety of benefits for the island (see below).

*RepA* and the preceding ORF *S004* constitute an operon that is regulated through the AcaCD-responsive promoter P*_S004_* by the AcaCD and FlhDC_SGI1_ activators encoded by IncC plasmids and SGI1, respectively. The plasmid pMSZ1016, composed of a resistance gene and the whole rep region of SGI1 including *oriV* and the *S004-repA* operon along with P*_S004_*, proved to be replicable in the presence of any FlhDC-like activators if they are in excess. However, the maintenance of pMSZ1016 required both activators when expressed under their native control systems of the IncC plasmid and SGI1, respectively. Therefore, SGI1 synchronizes its plasmid-like replication with the presence of the helper plasmid similarly to the timing of excision, i.e. the replication machinery is also triggered by the helper-encoded activator AcaCD. Thus, SGI1 is the first example for a helper-induced plasmid-like replication of an IME. This seems to be a good strategy, as a constitutive replication would be harmful to the host when the IME is integrated into the chromosome.

The comparison of search results in GenBank database with the rep region and the 27.4 kb backbone sequence of SGI1 showed that all the 122 retrieved SGI1 homologs having >60% coverage to the backbone sequence carry the homolog of SGI1-derived replicon including P*_S004_*, *S004, repA* and *oriV* in the same constitution as found in SGI1. This indicates the high conservation of this rep region in SGI1-family elements. The only exception is the chromosome of the *S. enterica* serovar Kentucky strain VNSEC0 (CP039439), where an SGI1-like element showing less than 40% coverage to the SGI1 backbone also contains the homolog of SGI1 rep region. A search for more divergent SGI1-related elements revealed two SGI1-like GIs sharing a similar structure to SGI1, but having a different *rep* gene (51). These elements, GI*Vch*O27–1 and GI*Sen*-26, identified in *V*. *cholerae* O27 and *S*. *enterica* serovar Muenster 26 strains, respectively, are also located at the 3′ end of *trmE* and show 94% identity to each other but have much lower sequence similarity to SGI1. Their *repA* is unrelated to that of the IncN2/N3 group and encodes a Rep-Primase family protein, whose homologs occur also in several SGI1-related elements found in *P. mirabilis*, *S. enterica* serovars and some other species (51). The *repA* of GI*Vch*O27–1 and GI*Sen*-26, like in SGI1, is preceded by an *S004* homolog whose putative protein product shares only 33% identity to S004 of SGI1. Although these *S004-rep* operons also appear to be driven by an AcaCD-responsive promoter, the different genetic context of their two genes (i. e., there is no overlap, instead, a ca. 200 bp space separates the STOP of *S004* and START of *repA*) suggests that *S004* and *repA* are not translationally coupled. Therefore the amount of RepA, and consequently, the copy number of the excised GIs are probably controlled in a different way than in SGI1.

The copy number of SGI1, similarly to the SGI1-derived replicon pMSZ1016, depends on the amount of RepA within a certain range (Figures 2, 5, Supplementary Figure S5, and Table 1). The β-galactosidase assays showed that both the promoter activity of P*_S004_* and the translation of RepA are proportional to the quantity of AcaCD or FlhDC_SGI1_ when present in excess and both activators are necessary if they are expressed from their original source (Figure 5). These results support that expression of RepA_SGI1_ and consequently, the copy number of SGI1 are under the double control of AcaCD and FlhDC_SGI1_. AcaCD proved to be a stronger activator on the AcaCD-responsive promoters of SGI1 than FlhDC_SGI1_ (33) and seems to be expressed from the helper plasmid in sufficient amount for triggering SGI1 excision, but not for inducing proper replication alone. To maintain the 4–8 SGI1 copies/cell, both AcaCD and FlhDC_SGI1_ are required in the amount provided by their own expression mechanisms (14,22), while in the absence of FlhDC_SGI1_, the island seems to be maintained only as a single copy plasmid (Figure 6B). Thus, SGI1 controls, at least partially, its copy number by expressing its own activator FlhDC_SGI1_.

Our results indicate that the copy number of SGI1 is controlled through the expression level of RepA_SGI1_. We have shown that in addition to the transcriptional activation of P*_S004_* by the two FlhDC-family activators, the other key element in this control mechanism is the translational coupling of *repA* and ORF *S004*. It has been reported that the expression of the *repA* gene cannot be measured with a β-galactosidase test plasmid where the *lacZ* is fused to the START codon of *repA* even in the presence of excess AcaCD (33). However, the analysis of the sequence near the *S004-repA* overlap uncovered an IR-motif, which can form a stem-loop structure on the *S004-repA* transcript. Such long-range RNA structures have a central role in controlling *repA* expression and regulation of copy number in numerous plasmid families (56). Using β-galactosidase test plasmids that were designed by taking into consideration the potential requirement for the intactness of this stem-loop structure, we have confirmed that the AcaCD- and FlhDC_SGI1_-controlled P*_S004_* drives the expression of RepA_SGI1_ (Figure 5). We have also shown that translation of S004 and RepA is tightly coupled as an early STOP mutation in ORF *S004S* prevents RepA expression and SGI1 replication and this mutation cannot be rescued by providing S004S protein *in trans*. This result supports that the S004 protein acts as a leader peptide, of which translation *in cis* is indispensable for the translation of RepA.

Proper expression of RepA was observed only when the IR motif remained intact (Figure 5), indicating the importance of the potential stem-loop structure in the *S004-repA* transcript (33). The IR overlaps the translation START site of *repA* along with the downstream STOP codon of *S004*, which are normally positioned close to each other. The putative stem-loop structure efficiently masks the Shine-Dalgarno sequence (SD) and the START codon of *repA* on the mRNA (note that RepA expression was not observed in the absence of S004 translation even using a strong promoter like the IPTG-induced P_tac_). However, when translating the C-terminus of S004 leader protein, the stalled ribosomes possibly resolve the stem-loop, which uncovers the translation START site of *repA*. This constitution ensures the reinitiation of the ribosome at the SD site of RepA. The early STOP codon in ORF *S004* prevents the ribosomes from reaching the stem-loop and thus the initiation of RepA translation fails. Similar translational coupling mechanisms are prevalent among plasmid replication systems and appear as a general evolutionary strategy in copy number control (56,57). The genetic constitution seen in the SGI1 rep region suggests that the IncN2/N3-related SGI1-derived replicon has also adopted such a control mechanism.

The stable vertical transfer of an IME is mostly based on the chromosomal integration, however, when the mobilization helper element appears, the IME has to give this safe position up for the benefits of horizontal transfer. The chance for the lateral transfer accompanies the transient destabilization of SGI1 when coexisting with the IncC helper plasmid (14). This negative effect of the helper is considerably reduced by the SGI1-encoded TA-system (16), however, it cannot entirely prevent SGI1 loss (14). Our results clearly indicate that plasmid-like replication of the excised circular SGI1 significantly contributes to its stability. It was obvious for all replication-deficient SGI1 mutants, which showed extremely high rate of segregation in the presence of the helper. Moreover, maintenance of SGI1 at ca. 6 copies per cell in the presence of the helper plasmid may have further important advantages. SGI1 has been suspected to encode for destabilizing functions against its helper plasmids (16,25). These might be more efficient when expressed from elevated number of SGI1 copies. The copy number of the Δ*flhDC*_SGI1_ mutant decreased to ca. 1 per cell when the helper was present and showed not only lower stability, but it was much less efficient in the displacement of the helper (Figure 6C). The SGI1-K variant lacking *flhDC*_SGI1_ was shown not to destabilize pRMH760 helper (25). These observations might be explained by the direct destabilizing effect of FlhDC_SGI1_ protein on the IncC plasmids. On the other hand, the fact that R55 appeared stable together with the FlhDC_SGI1_-producer plasmid pGMY6 in a 100-generation growth without selection (unpublished data) makes this explanation unlikely. Instead, we propose that the lower copy-number of the Δ*flhDC*_SGI1_ mutant better explains this phenomenon. Although our results also support that the SGI1 replication is linked to the incompatibility with IncC plasmids as suggested by (51), this effect is unlikely to be based on the direct effect of FlhDC_SGI1_ or the conflict of the replication machineries, as indicated by the compatibility of the SGI1-derived basic replicon with the IncC helper. We suggest instead that the increased dosage of a yet unidentified SGI1-encoded incompatibility factor (due to the elevated copy number of SGI1) serves as a better explanation for the efficient destabilization of the helper.

Outperforming the transfer of helper plasmid by SGI1 has been reported several times (22,25), and the role of SGI1-encoded T4SS components TraN_S_, TraG_S_ and TraH_S_ in remodelling the helper-encoded mating pore has been demonstrated. It seems that the ‘hybrid’ T4SS modified by the SGI1-encoded components favours SGI1 transfer over that of the helper plasmid and helps SGI1 to evade the helper-encoded entry exclusion (32). However, the fact that SGI1 transiently overreplicates the single-copy helper plasmid raises the possibility that merely the higher copy-number of SGI1 contributes to the more efficient transfer, which is further supported by the elevated expression of SGI1-genes involved in the transfer.

Interesting analogies can be seen in the interrelations of SGI1-IncC helpers and the viral satellites, the phage-inducible chromosomal island-like elements (PLE), and their target phages. PLE1–5 are chromosomal islands of *V. cholerae* whose excision is induced by ICP1 phage. While in SGI1, the RDF (Xis) expression is induced by the helper-encoded activator, the ICP1 helper phage itself provides the RDF (PexA) for PLE excision (58). Further similarities are that the excised PLE overreplicates its helper phage, outperforms the phage genome in packaging and consequently, highly reduces the spread of the helper phage (59). Thereby PLEs provide efficient defence for their host against its lytic phage invaders and ensure their own horizontal transfer at the expense of the helper phage (59,60). PLE also has its own replication origin and encodes a RepA resembling Gram+ plasmid replication initiation factors. Similarly to SGI1, the replication is helper-dependent as it is triggered by an unknown helper-encoded factor (59). Although the molecular mechanisms are different, PLEs also exploit their helper phages in multiple ways, which is performed by hijacking the helper-encoded proteins as in case of SGI1. Despite that the genetic background of these interventions seems completely different, the analogies suggest that there are common selection forces acting in the co-evolution of SGI1-like IMEs and PLEs with their respective helper elements.

SGI1 appears exceptional among IMEs as it is not simply an opportunistic hitch-hiker of its mobilization helpers, the IncA and IncC plasmids, but has been evolved to efficiently hijack the helper-encoded machinery to its own benefit, ensuring its most efficient vertical and horizontal transfer. Advantages over the helper plasmid are gained by multiple fine-tuned interventions into the crosstalk between SGI1 and its helper. This occurs mostly by transient expression of several SGI1 genes triggered by the helper-encoded AcaCD activator (22,14,32,31). Furthermore, SGI1 destabilizes its helper plasmid (25), reducing the time of co-habitation. Its evolutionary benefits possibly root in the fact that the helper also destabilizes SGI1 by triggering its excision (14). The helper-induced transient replication of SGI1 seems to be a refined adaptive trait, ensuring the more stable vertical transfer and more efficient horizontal transfer of SGI1 when the helper element appears. The diverse ways by which SGI1 exploits the IncA and IncC plasmids certainly played an important role in the outstanding success of SGI1-family elements in their distribution among pathogenic enteric bacteria.

## Supplementary Material

gkaa1257_Supplemental_Files
