# Supplementary material for: IncC helper dependent plasmid-like replication of Salmonella Genomic Island 1
Source: Nucleic Acids Res. 2021 Jan 6;49(2):832–46. doi: 10.1093/nar/gkaa1257 (PMC7826253; doi:10.1093/nar/gkaa1257)
Supplement: gkaa1257_Supplemental_Files [file gkaa1257_supplemental_files.zip › Supplementary materials_revised.docx]

**Additional Materials and Methods and Supplementary data**

**Supplementary Text S1.**

pGMY9: The Km^R^ derivative of pJKI1048 (1). The Sm^R^ gene was deleted with *Hin*dIII digestion and religation of the plasmid backbone.

pMSZ1003: The 1901-2161 bp (*oriV*) region of SGI1 was amplified from *E. coli* with SGI_oriVSNfor -SGI_oriVNBrev primers and the amplicon was cloned into the *Not*I site of pSG76-CS.

pMSZ1008: The 1901-3443 bp region of SGI1 was amplified with SGI_oriVSNfor - SGI_repNBrev primers and the amplicon was cloned into the *Not*I site of pSG76-CS (insert is in the opposite direction to the *cat*).

pMSZ1012: pJKI1048 was digested with *Dra*I-*Pst*I and the fragment containing the *rep*A of SGI1 under the control of P_tac_ was cloned into the *Sma*I-*Pst*I site of pMSZ1003. The *oriV* of R6K was deleted by *i-Sce*I and *Pst*I digestion and religation of the plasmid backbone after the protruding end was converted to blunt end using NEBNext® End Repair Module.

pMSZ1014: The *Eco*RI-*Bam*HI fragment of pJKI882 (2) was ligated into the *Eco*RI-*Bam*HI site of pJKI788 (2) leading to pMNI16. The *Nde*I-*Bam*HI fragment of pMNI16 was replaced by the *Nde*I-*Bam*HI fragment of pGMY9. The resulting plasmid, pMSZ1010, carried the 2162-3115 bp (*repA*) region of SGI1 under the control of P_tac_ joined to the Km^R^ gene. Finally, this assembled region was then cloned into the *Not*I site between the IS*10* ends in pJKI334 (2).

pMSZ1016: The 3280-3494 bp region of SGI1 was amplified with SGI_seq1for – S003promrevX primers and the amplicon was cloned into the *Xba*I-*Pst*I site of pMSZ1008 extending the rep-region of SGI1 and deleting the *oriV* region of R6K-based pSG76-CS. The ligated DNA was transformed into TG1Nal::*repA*_SGI1_ strain.

pMSZ1028: The 2970-3295 bp region of SGI1 was amplified with d004for - S004_stop primers, the amplicon was digested with *Pst*I-*Eco*RV and cloned into the *Pst*I-*Eco*RV site of pMSZ1016. This introduced an ochre (UAA) codon in *S004* ORF (AG to TA change at positions 3259-3260 of SGI1). The ligated DNA was transformed into TG1Nal::*repA*_SGI1_ strain.

pMSZ1030: The chromosomal *lacZ* fragment from *E. coli* W3350 was amplified using primers lacZ_fusionBfor – lacZ-Clarev and cloned into the *Xba*I-*Cla*I site of pJKI990 removing the 1-7 aa of the translated LacZ protein and exchanging the cloning site of plasmid.

pMSZ1032: The 3074-3494 bp rep-region of SGI1 was amplified with S003promrevX - S003fusionforB primers and the amplicon was cloned into the *Xba*I-*Bam*HI site of pMSZ1030.

pMSZ1034: The rep-region of pMSZ1028 harbouring an stop (UAA) codon in *S004* was amplified with S003promrevX - S003fusionforB primers and the amplicon was cloned into the *Xba*I-*Bam*HI site of pMSZ1030.

pMSZ1037: The protruding end of *Eco*RI digested pJKI1050 was converted to blunt end using NEBNext® End Repair Module and also digested with *Pst*I. The fragment containing the P_tac_ region was cloned into the *Pst*I and the filled *Xba*I site of pMSZ1032.

pMSZ1039: The *Bam*HI-*Pst*I fragment of pMSZ1037 was replaced by the *Bam*HI-*Pst*I fragment of pMSZ1034 harbouring an ochre (UAA) codon in *S004*.

pMSZ1040: The Km^R^ derivative of pJKI1050 (1). The Sm^R^ gene was deleted by *Hin*dIII digestion and religation of the plasmid backbone.

pMSZ1041: The junction fragment of circular SGI1 (attP: 42126-(42433/1)-496 bp) was amplified using primers RJ2X – LJ2XS and the amplicon was cloned into *Xba*I-*Sal*I site of pJKI336 (3), resulting in R6K-based delivery plasmid containing the the attP site with a Km^R^ gene in the transposable mini-Tn10 unit.

pMSZ1066:The *NdeI*-*Bam*HI fragment of pJKI1048 was cloned into the *Nde*I-*Bam*HI site of pET-16b.

pMSZ1070: The 1929-2123 bp (*oriV*) region of SGI1 was amplified with SGI_oriVforE - SGI_oriVrevBN primers and the amplicon was cloned into the *Eco*RI-*Bam*HI site of pSG76-CS.

pMSZ1071: The 1929-2083 bp (*oriV*) region of SGI1 was amplified with SGI_oriVforE -SGI_minimaloriVrevBN primers and the amplicon was cloned into the *Eco*RI-*Bam*HI site of pSG76-CS.

pMSZ1072: The 1929-2161 bp (*oriV*) region of SGI1 was amplified with SGI_oriVforE - SGI_ oriVNBrev primers and the amplicon was cloned into the *Eco*RI-*Bam*HI site of pSG76-CS.

pMSZ1073: The 1974-2123 bp (*oriV*) region of SGI1 was amplified with SGI_minimaloriVforE - SGI_oriVNBrev primers and the amplicon was cloned into the *Eco*RI-*Bam*HI site of pSG76-CS.

pMSZ1074: The 1974-2083 bp (*oriV*) region of SGI1 was amplified with SGI_minimaloriVforE - SGI_minimaloriVrevBN primers and the amplicon was cloned into the *Eco*RI-*Bam*HI site of pSG76-CS.

pMSZ1075: The 1974-2161 bp (*oriV*) region of SGI1 was amplified with SGI_minimaloriVforE - SGI_ oriVNBrev primers and the amplicon was cloned into the *Eco*RI-*Bam*HI site of pSG76-CS.

pMSZ1095: The *Eco*RI-*Bam*HI fragment of pMSZ1070 was cloned into the *Eco*RI-*Bam*HI site of pBluescriptIISK leading to the minimal *oriV* plasmid required for *in vivo* replication of SGI1.

pMSZ1098: The *Eco*RI-*Bam*HI fragment of pMSZ1074 was cloned into the *Eco*RI-*Bam*HI site of pBluescriptIISK resulting in plasmid carrying all RepA binding sites of SGI1.

pMSZ1113: The 1901-2083 bp (*oriV*) region of SGI1 was amplified with SGI_ fulloriVforE - SGI_minimaloriVrevBN primers and the amplicon was cloned into the *Eco*RI-*Bam*HI site of pSG76-CS.

pMSZ1114: The 1901-2123 bp (*oriV*) region of SGI1 was amplified with SGI_ fulloriVforE - SGI_oriVNBrev primers and the amplicon was cloned into the *Eco*RI-*Bam*HI site of pSG76-CS.

pMSZ1120: The SGI_oriV up and down primer pair was annealed and cloned into the the *Xba*I-*Pst*I site of pBluescript II SK resulting in plasmid containing a single binding site of RepA_SGI1_.

pMSZ1164: The 1901-2030 bp (*oriV*) region of SGI1 was amplified with SGI_ fulloriVforE - SGI_oriVrev2030BN primers and the amplicon was cloned into the *Eco*RI-*Bam*HI site of pSG76-CS.

pMSZ1165: The 1901-2056 bp (*oriV*) region of SGI1 was amplified with SGI_ fulloriVforE - SGI_oriVrev2056BN primers and the amplicon was cloned into the *Eco*RI-*Bam*HI site of pSG76-CS.

**Table S1.** Oligonucleotide primers used.

| Name | Sequence (5’→3’) | References |
| --- | --- | --- |
| SGI_oriVSNfor | cttcgtcgacgcggccgcaagttattaacgcctgaacatggttc | this work |
| SGI_oriVNBrev | tcgcggccgcggatcccttaataaccggccaatgtgcc | this work |
| sgiPxisdelfor1 | caccagttattgatagacctagtttatgagagacagctaccaatcctCATATGAATATCCTCCTTAGTTC | (4) |
| RJ2X | gctctagacgctcgaagaggtagagcag | this work |
| LJ2XS | gctctagagtcgacgcaagttttactctgtcttccag | this work |
| sgi006delfor | aattagcatcgctatttgcccttttgcggcatacgcggatgtattcaCATATGAATATCCTCCTTAGTTC | (4) |
| sgi007delrev | tagtttcaatcaatctgggccgcagaaaaaaaggtaaggaggactactgaGTGTAGGCTGGAGCTGCTTC | (4) |
| delflhDCfor | gagtcttggctatctatcgcaaccttcgtgatttgtgaggggggcggaatGTGTAGGCTGGAGCTGCTTC | (4) |
| delflhDCrev | cgcaaagtttgggcgctcatcttctggtccgaaatgtcatagtctactCATATGAATATCCTCCTTAGTTC | (4) |
| LJ3 | aacctaaccataagagaacttcc | this work |
| RJ5 | gatcagggagaattcgagtagag | this work |
| attsgirev2 | tgacagtcagaatgcggcttcg | this work |
| attsgirev3 | ctggagaaaatccgccccagc | this work |
| attsgifor2 | caacatggaaggcggcttcctg | this work |
| pxisseqrev | tgcgcgagcagtttggtcaag | this work |
| SGI_S003delfor | tgcaaaacaataacataaaaaaaccggcacattggccggttattaagCATATGAATATCCTCCTTAGTTC | this work |
| SGI_S003delrev | agttggaggcatggcttgattcccgcagggtaagtaatgaagccatttagGTGTAGGCTGGAGCTGCTTC | this work |
| SGI_S004-005delfor | atcattgatttactggagaaattttgagggtgaacccccgtaaacatCATATGAATATCCTCCTTAGTTC | this work |
| SGI_S004-005delrev | acgacaaagcgcgtgaaatctttcctgacttggatggcaatggtggttgaGTGTAGGCTGGAGCTGCTTC | this work |
| SGI_oriVforE | gcgaattcctataggtatctttataggatttttttaaaggc | this work |
| SGI_oriVrevBN | gcggatccgcggccgctgttttgcaaagttgtggaaaac | this work |
| SGI_minimaloriVrevBN | gcggatccgcggccgcttatccacgcataatcgcc | this work |
| SGI_minimaloriVforE | gcgaattctttagggggggggaaattatg | this work |
| SGI_fulloriVforE | gcgaattcaagttattaacgcctgaacatggttc | this work |
| SGI_oriVrev2056BN | gcggatccgcggccgctttacgcataattacccccctgac | this work |
| SGI_oriVrev2030BN | gcggatccgcggccgcctgacgagctgcgcataatc | this work |
| SGI_oriVup | ggggggtaattatgcgtaat | this work |
| SGI_oriVdown | acgtccccccattaatacgcattagatc | this work |
| SGI_repNBrev | cgggatccgcggccgcttgtgtcggaataaatcgcctag | this work |
| SGI_seq1for | gagatcatctgcaggtgactgtaatc | this work |
| SGI_seq1rev | aactgcagtggttgggtatacttcagc | this work |
| S003_BXhrev | aactcgagggatcctcaattacggtatggaatcggtgg | (1) |
| S003promrevX | tttctagaggcaatggtggttgatgtttatc | this work |
| lacZ-Clarev | aaccaccacgctcatcgataatttc | (4) |
| lacZ_fusionBfor | gctctagactgcagcccggggaattcggatccctggccgtcgttttacaacgtcg | this work |
| S003fusionforB | gcggatccatccgcctgatactgagcaag | this work |
| SGI_rev3597 | tttctagatgaaagtttttgtgtcggaataaatc | this work |
| SGI_rev3611 | tttctagaaactttcacatgtgaaagtttttgtgtcgg | this work |
| SGI_rev3629 | tttctagagttgatgtttatcaatgagaaactttc | this work |
| S004promforNc | gcccatggatttctccagcatcatcattgat | (1) |
| SGI_repfor | tgaggcttggattggtaacagc | this work |
| SGI_reprev | caaactgctcgcgcaaattac | this work |
| S004_stop | acctgcagatgatctctgtgtatgaggtgactgcc**ta**acttaaaatc | this work |
| d004for | tcattgggcgagggtggatctg | this work |

Restriction sites are underlined, single mutations are shown as bold, uppercase indicate the 3’ part of KO oligos annealing to the template plasmid pKD3 and pKD4.

| *E. coli* Strains | Genotype or relevant features*^a^* | References |
| --- | --- | --- |
|  |  |  |
| ME6266 | F^-^ *polA*11 *purE* *trp lys proC* *leu thi lacZ* *xyl ara tonA* *tsx str* | Shigen (ME) collection* |
| ME7772 | F^-^ *metE purE* *trp lys proC* *leu thi lacZ* *xyl ara tonA* *tsx str* | Shigen (ME) collection* |
| TG1 | *supE hsd*Δ*5 thi*Δ(*lac-proAB*) F’[*traD*36 *proAB^+^ lacI*^q^ *lacZ*ΔM15] | (5) |
| TG1Nal | Nal^R^ derivative of TG1 | (6) |
| TG1Nal::*repA*_SGI1_ | TG1Nal::[ miniTn*10*::*rep*A_SGI1_-Km^R^] containing the 2162-3115 bp SGI1 region (ORF *S003*) under the control of P*_tac_* promoter and *rrnB* terminator, Nal^R^Km^R^ | this work |
| TG1Nal::SGI1-C | TG1Nal containing SGI1-C variant integrated into the *E. coli thdF*, Nal^R^Sm^R^SpSul^R^ | (4) |
| TG1Nal::SGI1-C^Δ^*^oriV^* | TG1Nal containing the deletion mutant of SGI1-C, in which the *oriV* (1908-2161 bp) region was deleted, Nal^R^Sm^R^Sp^R^Sul^R^ | this work |
| TG1Nal::SGI1-C/R55^ΔTn^*^6187^* | TG1Nal containing SGI1-C and R55^ΔTn^*^6187^*, Nal^R^Sm^R^Sp^R^Sul^R^Cm^R^Flo^R^ | this work |
| TG1Nal/R16a | TG1Nal containing R16a, Nal^R^Km^R^Ap^R^Sul^R^ | (7) |
| TG1Nal::*attP*_SGI1_ | TG1Nal::[ miniTn*10*::*attP*_SGI1_-Km^R^] containing the junction fragment of circular SGI1 (*attP*: 42126-(42433/1)-496 bp), Nal^R^Km^R^ | this work |
| TG1Nal::SGI1-C/R16a | TG1Nal::SGI1-C containing R16a, Nal^R^Sm^R^Sp^R^Sul^R^Km^R^Ap^R^ | (4) |
| TG1Nal::SGI1-C/R16a^Δ^*^acaCD^* | TG1Nal::SGI1-C containing *acaCD* KO mutant R16a, Nal^R^Sm^R^Sp^R^Sul^R^Km^R^Ap^R^ | (1) |
| TG1Nal::SGI1-C^Δ^*^flhDC^* | TG1Nal containing the *flhDC* KO mutant SGI1-C, in which the ORF *S006-007* (6339-7482 bp) region was deleted, Nal^R^Sm^R^Sp^R^Sul^R^ | this work |
| TG1Nal::SGI1-C^Δ^*^flhDC^*/R16a | TG1Nal containing SGI1-C^Δ^*^flhDC^* and R16a, Nal^R^Sm^R^Sp^R^Sul^R^Km^R^Ap^R^ | this work |
| TG1Nal::SGI1-C^Δ^*^oriV^*/R16a | TG1Nal containing SGI1-C^Δ^*^oriV^* and R16a, Nal^R^Sm^R^Sp^R^Sul^R^Km^R^Ap^R^ | this work |
| TG1Nal::SGI1-C^Δ^*^repA^* | TG1Nal containing the *repA* KO mutant SGI1-C, in which the ORF *S003* (2162-3101 bp) region was deleted, Nal^R^Sm^R^Sp^R^Sul^R^ | this work |
| TG1Nal::SGI1-C^Δ^*^repA^*/R16a | TG1Nal containing SGI1-C^Δ^*^repA^* and R16a, Nal^R^Sm^R^Sp^R^Sul^R^Km^R^Ap^R^ | this work |
| TG1Nal::SGI1-C^ΔP^*^S004^* | TG1Nal containing the deletion mutant SGI1-C, in which the promoter region of *repA* (3393-3479 bp) was deleted, Nal^R^Sm^R^Sp^R^Sul^R^ | this work |
| TG1Nal::SGI1-C^ΔP^*^S004^*/R16a | TG1Nal containing SGI1-C^ΔP^*^S004^* and R16a, Nal^R^Sm^R^Sp^R^Sul^R^Km^R^Ap^R^ | this work |
| TG1Nal::SGI1-C^Δrep_region^ | TG1Nal containing the deletion mutant of SGI1-C, in which the rep-region between *xis* and ORF *S005* (1869-3479 bp) was deleted, Nal^R^Sm^R^Sp^R^Sul^R^ | this work |
| TG1Nal::SGI1-C^Δrep_region^/R16a | TG1Nal containing TG1Nal::SGI1-C^Δrep_region^ and R16a, Nal^R^Sm^R^Sp^R^Sul^R^Km^R^Ap^R^ | this work |
| TG1Nal::SGI1-C^Δrep_region^/R16a^Δ^*^acaCD^* | TG1Nal containing TG1Nal::SGI1-C^Δrep_region^ and *acaCD* KO mutant R16a, Nal^R^Sm^R^Sp^R^Sul^R^Km^R^Ap^R^ | this work |
| TG1Nal::SGI1-C^Δrep_region^ *^+^*^Δ^*^flhDC^*/ | TG1Nal containing the deletion mutant of SGI1-C, in which the rep-region between *xis* and ORF *S005* (1869-3479 bp) and the ORF *S006-007* (6339-7482 bp) region were deleted, Nal^R^Sm^R^Sp^R^Sul^R^Km^R^ | this work |
| TG1Nal::SGI1-C^Δrep_region^ *^+^*^Δ^*^flhDC^*/R16a | TG1Nal containing TG1Nal::SGI1-C^Δrep_region^*^+^*^Δ^*^flhDC^* and R16a, Nal^R^Sm^R^Sp^R^Sul^R^Km^R^Ap^R^ | this work |
| TG1Nal::SGI1-C^Δrep_region^/R55^ΔTn^*^6187^* | TG1Nal containing TG1Nal::SGI1-C^Δrep_region^ and R55^ΔTn^*^6187^*, Nal^R^Sm^R^Sp^R^Sul^R^Cm^R^Flo^R^ | this work |
| TG1Nal::SGI1-C^Δrep_region^ *^+^*^Δ^*^flhDC^*/R55^ΔTn^*^6187^* | TG1Nal containing TG1Nal::SGI1-C^Δrep_region^*^+^*^Δ^*^flhDC^* and R55^ΔTn^*^6187^*, Nal^R^Sm^R^Sp^R^Sul^R^Km^R^Cm^R^Flo^R^ | this work |
| TG1Nal::SGI1-C^Δrep_region^/R55^ΔTn^*^6187+^*^Δ^*^acaCD^* | TG1Nal containing TG1Nal::SGI1-C^Δrep_region^ and R55^ΔTn^*^6187+^*^Δ^*^acaCD^*, Nal^R^Sm^R^Sp^R^Sul^R^Cm^R^Flo^R^Km^R^ | this work |
| TG2 | *supE hsd*Δ*5 thi*Δ(*lac-proAB*)∆(*srl-recA*)306::Tn*10*(Tc^R^) F’[*traD*36 *proAB^+^ lacI*^q^ *lacZ*ΔM15] | (8) |
| TG90 | *pcn* B80 *zad*::*Tn*10 (Tc^R^) derivative of TG1 | (9) |
| TG90/R55^ΔTn^*^6187^* | TG90 containing R55^ΔTn^*^6187^*, Tc^R^Cm^R^Flo^R^ | this work |
| TG90/R16a | TG90 containing R16a, Tc^R^Km^R^Ap^R^Sul^R^ | this work |
| Tuner (DE3) | F^-^ *omp*T*hsdS*_B_(r_B_^-^ m_B_^-^) *gal dcm lacY*1 (DE3) | Novagen |
| S17-1 λpir | S17-1 λ*pir*, a λ lysogen derivative of S17-1 (*pro* *thi* *recA* *hsdR* (r- m+) Tpr Smr Kms [Ω RP4-2-Tc::Mu-Km::Tn7]) expressing Π protein from pir gene of R6K | (10) |

**Table S2.** Bacterial strains used in this study

***^a^*** Ap, ampicillin; Cm, chloramphenicol;Flo, florfenicol; Gm, gentamicin; Km, kanamycin; Nal, nalidixic acid; Rif, rifampicin; Sm, ; Su, sulphonamides; Tc, tetracycline; ^R/S^, resistant/sensitive

*source: <https://shigen.nig.ac.jp/ecoli/strain/>

**Table S3.** Relevant features of plasmids used in this study.

| Name | Relevant features*^a^* | References |
| --- | --- | --- |
| pCP20 | Ap^R^, Cm^R^, thermo-inducible FLP recombinase expression (λ pR::FLP), temperature-sensitive pSC101 replication system, λ cI857 | (11) |
| pCU999 | Km^R^ pCU1 derivative | (12) |
| pGMY6 | Km^R^ pJKI391 derivative containing the *flhDC*_SGI1_ genes of SGI1 (6336-7483 bp) under the control of P_tac_ promoter | (1) |
| pGMY9 | Km^R^ pJKI391 derivative containing *repA*_SGI1_ (ORF *S003*) of SGI1 (2162-3115 bp) under the control of P_tac_ and *rrnB* terminator | this work |
| pJKI88 | Km^R^ p15A cloning vector | (13) |
| pJKI391 | pJKI88 derivative expression vector containing P_tac_ followed by a MCS and *lacI^q^* gene | (4) |
| pJKI828 | Km^R^ pJKI88 derivative containing the whole putative operon of *acaCD* (166138-169131 bp of R55) | (4) |
| pJKI888 | Km^R^ pJKI391 derivative containg the *acaCD* genes (167704-168860 bp of R55) under the control of P_tac_ | (4) |
| pJKI990 | Ap^R^, Sm^R^, Sp^R^ cloning vector for β-gal assays containing the rrnB terminator, cloning sites, and the promoterless *lac*Z gene | (4) |
| pJKI1050 | Sm/Sp^R^ derivative of pJKI391 containing ORF *S004*_S_ of SGI1 under the control of P_tac_ and *rrnB* terminator | (1) |
| pKD3 | Cm^R^, Ap^R^ r6kγ-based PCR template plasmid for one-step recombination gene-KO | (14) |
| pKD4 | Km^R^, Ap^R^ r6kγ-based PCR template plasmid for one-step recombination gene-KO | (14) |
| pKD46 | Ap^R^, ara-inducible expression of λ Red recombinase, temperature-sensitive pSC101 replication system | (14) |
| pMSZ965 | Ap^R^, Sm^R^, Sp^R^ pJKI990 derivative tester plasmid containing P*_repA_* fragment (3332-3494 bp of SGI1) | (1) |
| pMSZ1003 | Cm^R^ pSG76-CS derivative harbouring 1901-2161 bp region of SGI1 | this work |
| pMSZ1012 | Cm^R^ pSG76-CS derivative harbouring the SGI1-derived basic replicon (*oriV*_SGI1_+P_tac_::*repA*_SGI1)_, but lacking the R6Kγ replication origin | this work |
| pMSZ1014 | Ap^R^,Km^R^ pJKI334 (2) derivative R6K-based delivery plasmid containing the *repA*_SGI1_ (2162-3115 bp) under the control of P_tac_ with a Km^R^ gene in the transposable mini-Tn*10* unit. | this work |
| pMSZ1016 | Cm^R^ pSG76-CS derivative harbouring the intact rep-region of SGI1 (1901-3494 bp), but lacking the R6Kγ replication origin | this work |
| pMSZ1028 | pMSZ1016 derivative obtained by introducing AG to TA change at positions 3259-3260 resulting in an early stop codon (UAA) in *S004*. | this work |
| pMSZ1030 | Ap^R^, Sm^R^, Sp^R^ cloning vector for β-gal assays containing the rrnB terminator, cloning sites, and the 5’ truncated *lac*Z gene | this work |
| pMSZ1032 | Ap^R^, Sm^R^, Sp^R^ pMSZ1030 derivative tester plasmid containing the 3074-3494 bp SGI1 region | this work |
| pMSZ1034 | pMSZ1032 derivative obtained by introducing AG to TA change at positions 3259-3260 resulting in an early stop codon (UAA) in *S004*. | this work |
| pMSZ1037 | pMSZ1032 derivative, in which S004 ORF is under the control P_tac_ | this work |
| pMSZ1039 | pMSZ1034 derivative, in which S004 ORF is under the control P_tac_ | this work |
| pMSZ1040 | Km^R^ derivative of pJKI1050 containing ORF *S004S* of SGI1 under the control of P_tac_ and *rrnB* terminator (Km^R^ gene was restored in pJKI1050 by deletion of Sm^R^/Sp^R^ cassette by HindIII digestion and religation). | this work |
| pMSZ1041 | Ap^R^,Km^R^ pJKI336 (15) derivative R6K-based plasmid containing the attP site of circular SGI1 (42126-(42433/1)-496 bp) with a Km^R^ gene in the transposable mini-Tn*10* unit. | this work |
| pMSZ1066 | pET16b (Novagen) derivative containing *repA*_SGI1_ genes under the control of P_T7_ | this work |
| pMSZ1070 | Cm^R^ pSG76-CS derivative harbouring 1929-2123 bp region of *oriV*_SGI1_ | this work |
| pMSZ1071 | Cm^R^ pSG76-CS derivative harbouring 1929-2083 bp region of *oriV*_SGI1_ | this work |
| pMSZ1072 | Cm^R^ pSG76-CS derivative harbouring 1929-2161 bp region of *oriV*_SGI1_ | this work |
| pMSZ1073 | Cm^R^ pSG76-CS derivative harbouring 1974-2123 bp region of *oriV*_SGI1_ | this work |
| pMSZ1074 | Cm^R^ pSG76-CS derivative harbouring 1974-2083 bp region of *oriV*_SGI1_ | this work |
| pMSZ1075 | Cm^R^ pSG76-CS derivative harbouring 1974-2161 bp region of *oriV*_SGI1_ | this work |
| pMSZ1095 | Ap^R^ pBluescript II-SK (16) derivative harbouring 1929-2123 bp region of *oriV*_SGI1_ | this work |
| pMSZ1098 | Ap^R^ pBluescript II-SK derivative harbouring 1974-2083 bp region of *oriV*_SGI1_ | this work |
| pMSZ1113 | Cm^R^ pSG76-CS derivative harbouring 1901-2083 bp region of *oriV*_SGI1_ | this work |
| pMSZ1114 | Cm^R^ pSG76-CS derivative harbouring 1901-2123 bp region of *oriV*_SGI1_ | this work |
| pMSZ1120 | Ap^R^ pBluescript II-SK derivative harbouring 2035-2055 bp region of *oriV*_SGI1_ | this work |
| pMSZ1164 | Cm^R^ pSG76-CS derivative harbouring 1901-2030 bp region of *oriV*_SGI1_ |  |
| pMSZ1165 | Cm^R^ pSG76-CS derivative harbouring 1901-2056 bp region of *oriV*_SGI1_ | this work |
| pSG76-CS | Cm^R^ R6K based cloning vector | (17) |
| R16a | Ap^R^Km^R^Sul^R^ | (18) |
| R16a^Δ^*^acaCD^* | *acaCD* KO mutant of R16a, Ap^R^Km^R^Sul^R^ | (1) |
| R55^ΔTn^*^6187^* | R55 derivative, in which Tn*6187* was deleted, Cm^R^,Flo^R^Sul^R^Ap^S^Km^S^Gm^S^ | (2) |
| R55^ΔTn^*^6187+^*^Δ^*^acaCD^* | *acaCD* KO mutant of R55 ^ΔTn^*^6187^*, Cm^R^,Flo^R^Sul^R^Km^R^ | this work |

Supplementary references

1. Murányi,G., Szabó,M., Olasz,F. and Kiss,J. (2016) Determination and Analysis of the Putative AcaCD-Responsive Promoters of Salmonella Genomic Island 1. *PLoS One*, **11**, e0164561.

2. Kiss,J., Szabó,M., Hegyi,A., Douard,G., Praud,K., Nagy,I., Olasz,F., Cloeckaert,A. and Doublet,B. (2019) Identification and Characterization of oriT and Two Mobilization Genes Required for Conjugative Transfer of Salmonella Genomic Island 1. *Front. Microbiol.*, **10**, 1–16.

3. Imre,A., Olasz,F., Kiss,J. and Nagy,B. (2006) A novel transposon-based method for elimination of large bacterial plasmids. *Plasmid*, **55**.

4. Kiss,J., Papp,P.P., Szabó,M., Farkas,T., Murányi,G., Szakállas,E. and Olasz,F. (2015) The master regulator of IncA/C plasmids is recognized by the Salmonella Genomic island SGI1 as a signal for excision and conjugal transfer. *Nucleic Acids Res.*, **43**, 8735–8745.

5. Gibson,T.J. (1984) Studies on the Epstein-Barr virus genome. Thesis.

6. Kiss,J., Nagy,B. and Olasz,F. (2012) Stability, entrapment and variant formation of Salmonella genomic island 1. *PLoS One*, **7**, e32497.

7. Szabó,M., Nagy,T., Wilk,T., Farkas,T., Hegyi,A., Olasz,F. and Kiss,J. (2016) Characterization of Two Multidrug-Resistant IncA/C Plasmids from the 1960s by Using the MinION Sequencer Device. *Antimicrob. Agents Chemother.*, **60**, 6780–6786.

8. Sambrook,J., Fritsch,E.F. and Maniatis,T. (1989) Molecular Cloning: A Laboratory Manual. Cold Spring Harbor Laboratory Press, Cold Spring Harbor, NY.

9. Gonzy-Treboul,G., Karmazyn-Campelli,C. and Stragier,P. (1992) Developmental regulation of transcription of the Bacillus subtilis ftsAZ operon. *J. Mol. Biol.*, **224**, 967–979.

10. Simon,R., Priefer,U. and Pühler,A. (1983) A Broad Host Range Mobilization System for In Vivo Genetic Engineering: Transposon Mutagenesis in Gram Negative Bacteria. *Bio/Technology*, **1**, 784–791.

11. Cherepanov,P.P. and Wackernagel,W. (1995) Gene disruption in Escherichia coli: TcR and KmR cassettes with the option of Flp-catalyzed excision of the antibiotic-resistance determinant. *Gene*, **158**, 9–14.

12. Papp,P.P. and Iyer,V.N. (1995) Determination of the binding sites of RepA, a replication initiator protein of the basic replicon of the IncN group plasmid pCU1. *J. Mol. Biol.*, **246**, 595–608.

13. Kiss,J. and Olasz,F. (1999) Formation and transposition of the covalently closed IS 30 circle : the relation between tandem dimers and monomeric circles. *Mol. Microbiol.*, **34**, 37–52.

14. Datsenko,K.A. and Wanner,B.L. (2000) One-step inactivation of chromosomal genes in Escherichia coli K-12 using PCR products. *Proc. Natl. Acad. Sci. U. S. A.*, **97**, 6640–5.

15. Herrero,M., De Lorenzo,V. and Timmis,K.N. (1990) Transposon vectors containing non-antibiotic resistance selection markers for cloning and stable chromosomal insertion of foreign genes in gram-negative bacteria. *J. Bacteriol.*, **172**, 6557–6567.

16. Alting-Mees,M.A. and Short,J.M. (1989) pBluescript II: gene mapping vectors. *Nucleic Acids Res.*, **17**, 9494–9494.

17. Kolisnychenko,V., Plunkett,G., Herring,C.D., Fehér,T., Pósfai,J., Blattner,F.R. and Pósfai,G. (2002) Engineering a reduced Escherichia coli genome. *Genome Res.*, **12**, 640–7.

18. Chabbert,Y.A., Scavizzi,M.R., Witchitz,J.L., Gerbaud,G.R. and Bouanchaud,D.H. (1972) Incompatibility Groups and the Classification of f- Resistance Factors. *J. Bacteriol.*, **112**, 666–675.
