# Supplementary figures and images for: IncC helper dependent plasmid-like replication of Salmonella Genomic Island 1
Source: Nucleic Acids Res. 2021 Jan 6;49(2):832–46. doi: 10.1093/nar/gkaa1257 (PMC7826253; doi:10.1093/nar/gkaa1257)

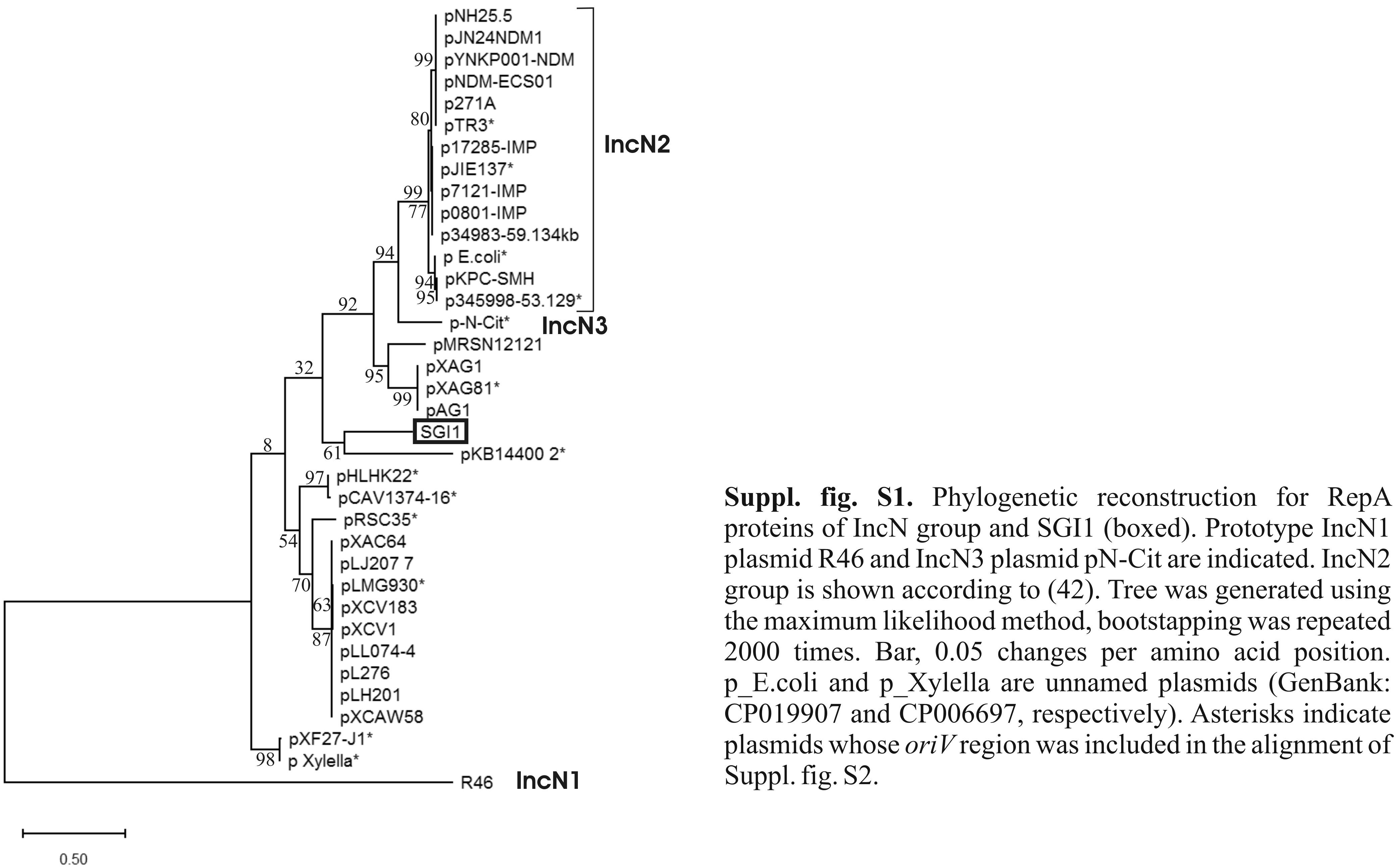

Supplement: gkaa1257_Supplemental_Files [file gkaa1257_supplemental_files.zip › fig S1.tif]

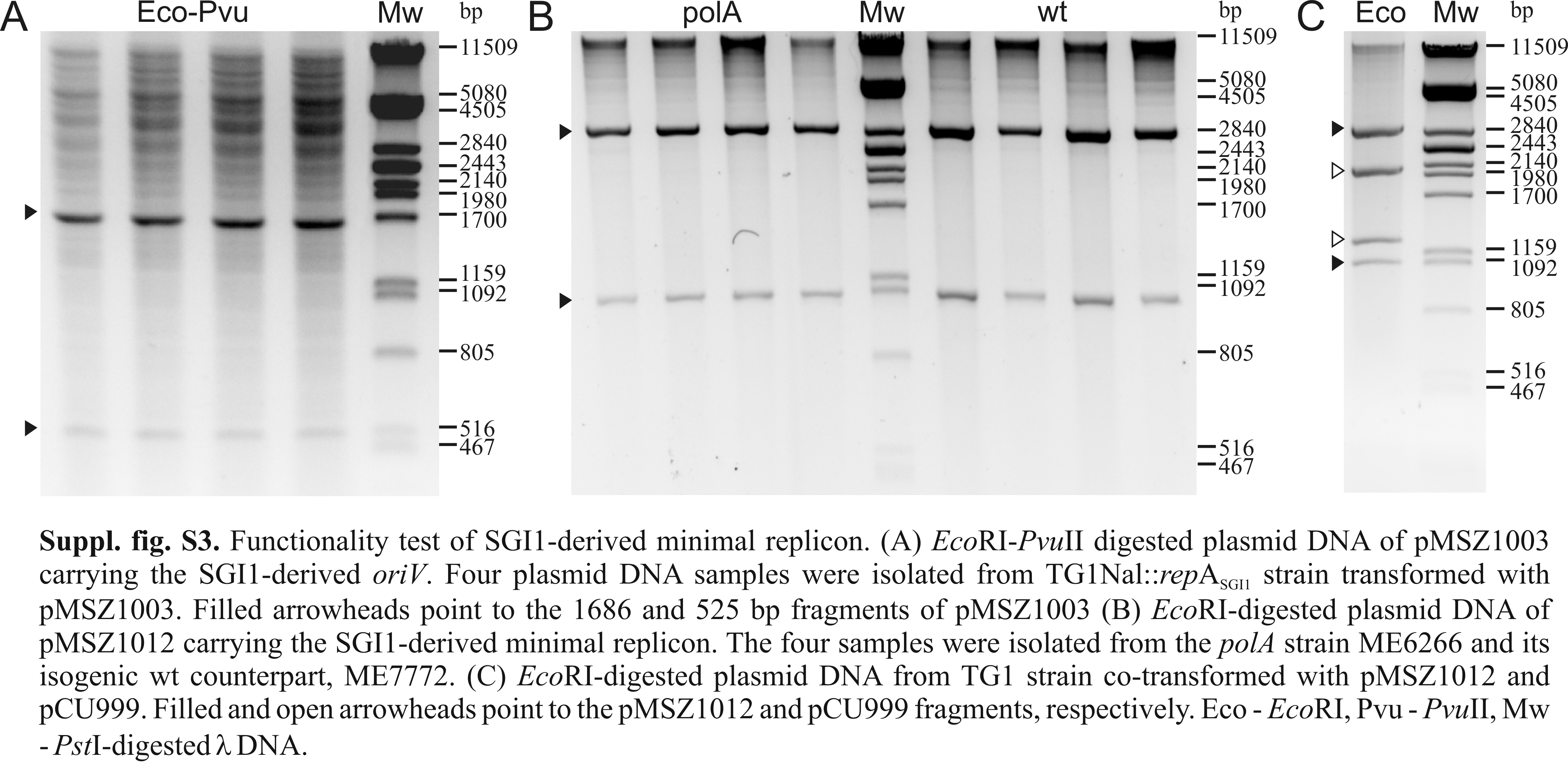

Supplement: gkaa1257_Supplemental_Files [file gkaa1257_supplemental_files.zip › fig S3.tif]

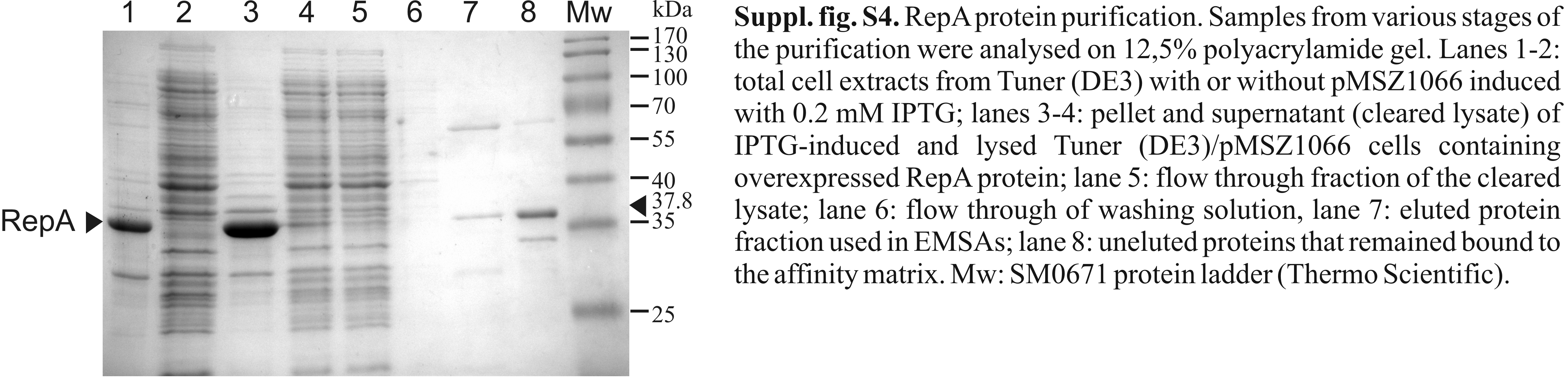

Supplement: gkaa1257_Supplemental_Files [file gkaa1257_supplemental_files.zip › fig S4.tif]

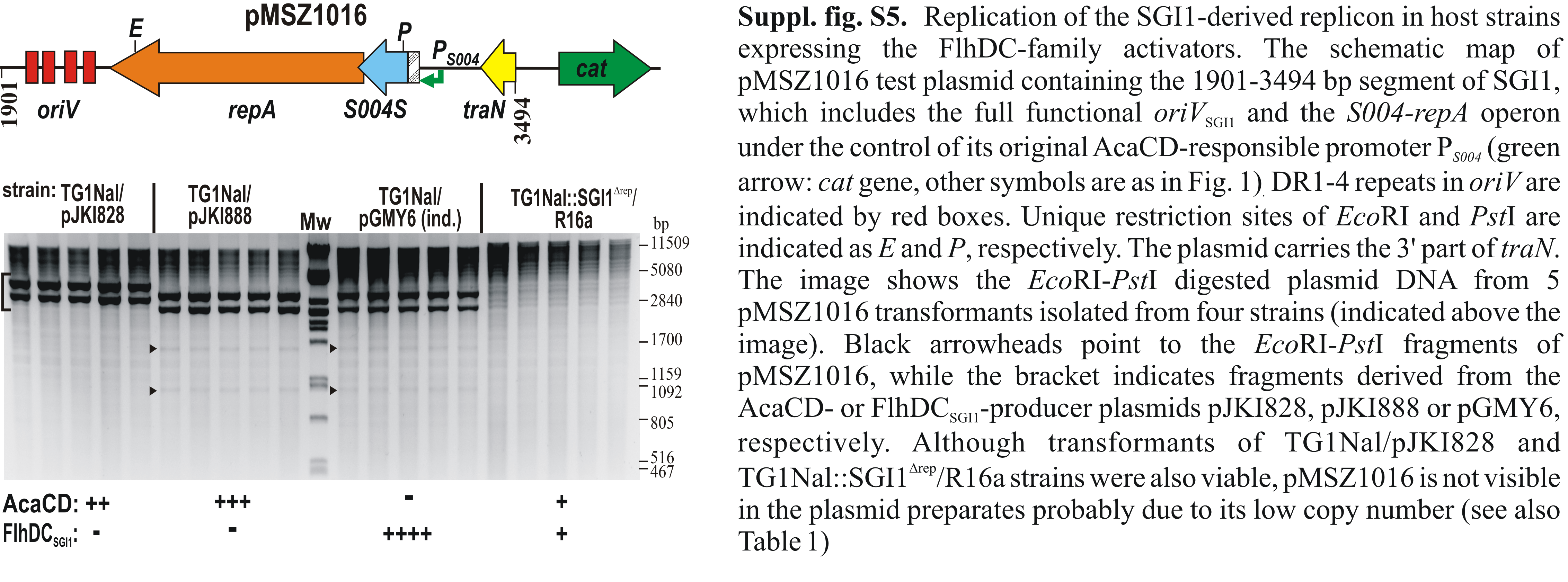

Supplement: gkaa1257_Supplemental_Files [file gkaa1257_supplemental_files.zip › fig S5.tif]

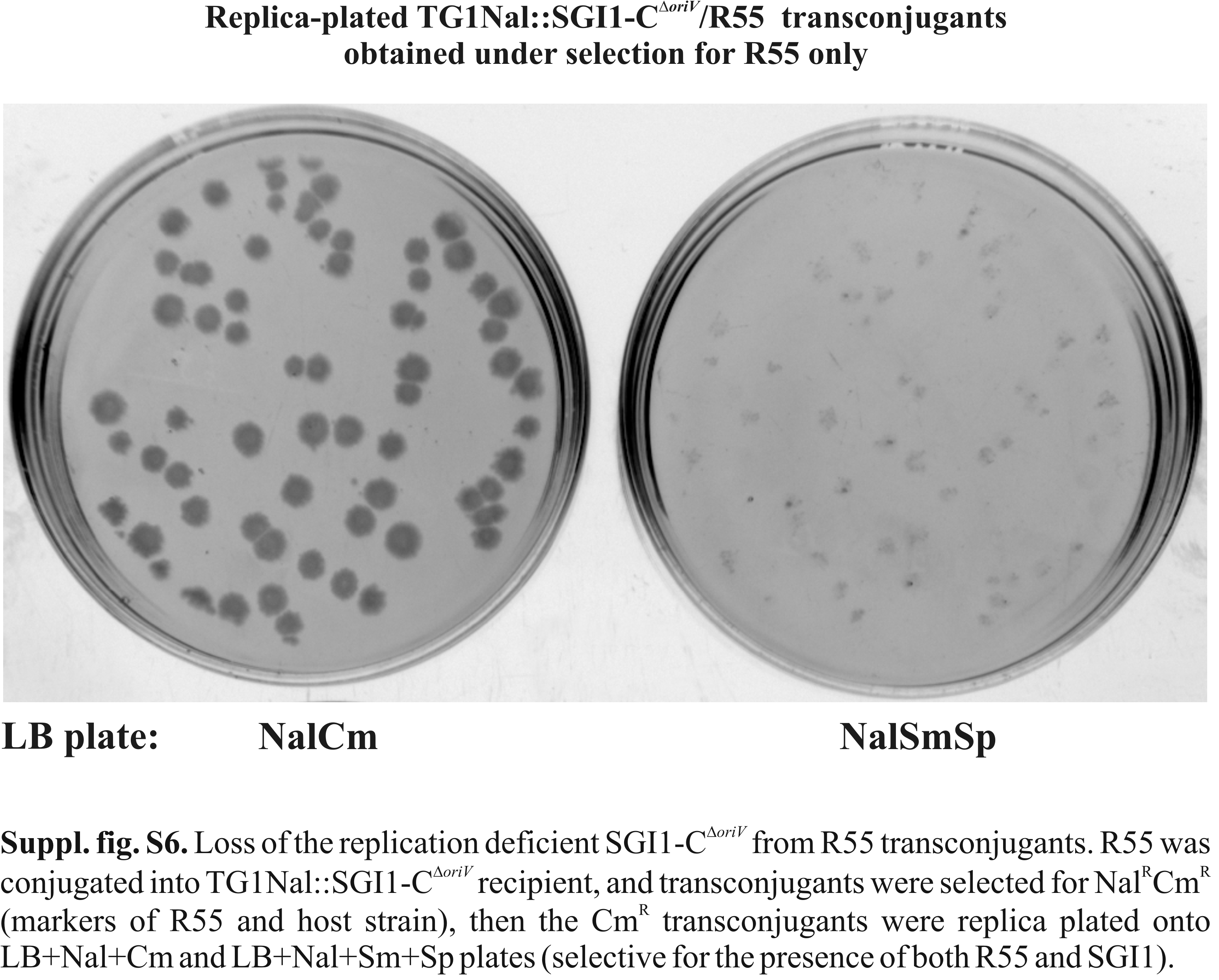

Supplement: gkaa1257_Supplemental_Files [file gkaa1257_supplemental_files.zip › fig S6.tif]
