# Supplementary material for: IncC helper dependent plasmid-like replication of Salmonella Genomic Island 1
Source: Nucleic Acids Res. 2021 Jan 6;49(2):832–46. doi: 10.1093/nar/gkaa1257 (PMC7826253; doi:10.1093/nar/gkaa1257)
Supplement: gkaa1257_Supplemental_Files [file fig S2.pdf › fig S2.pdf]

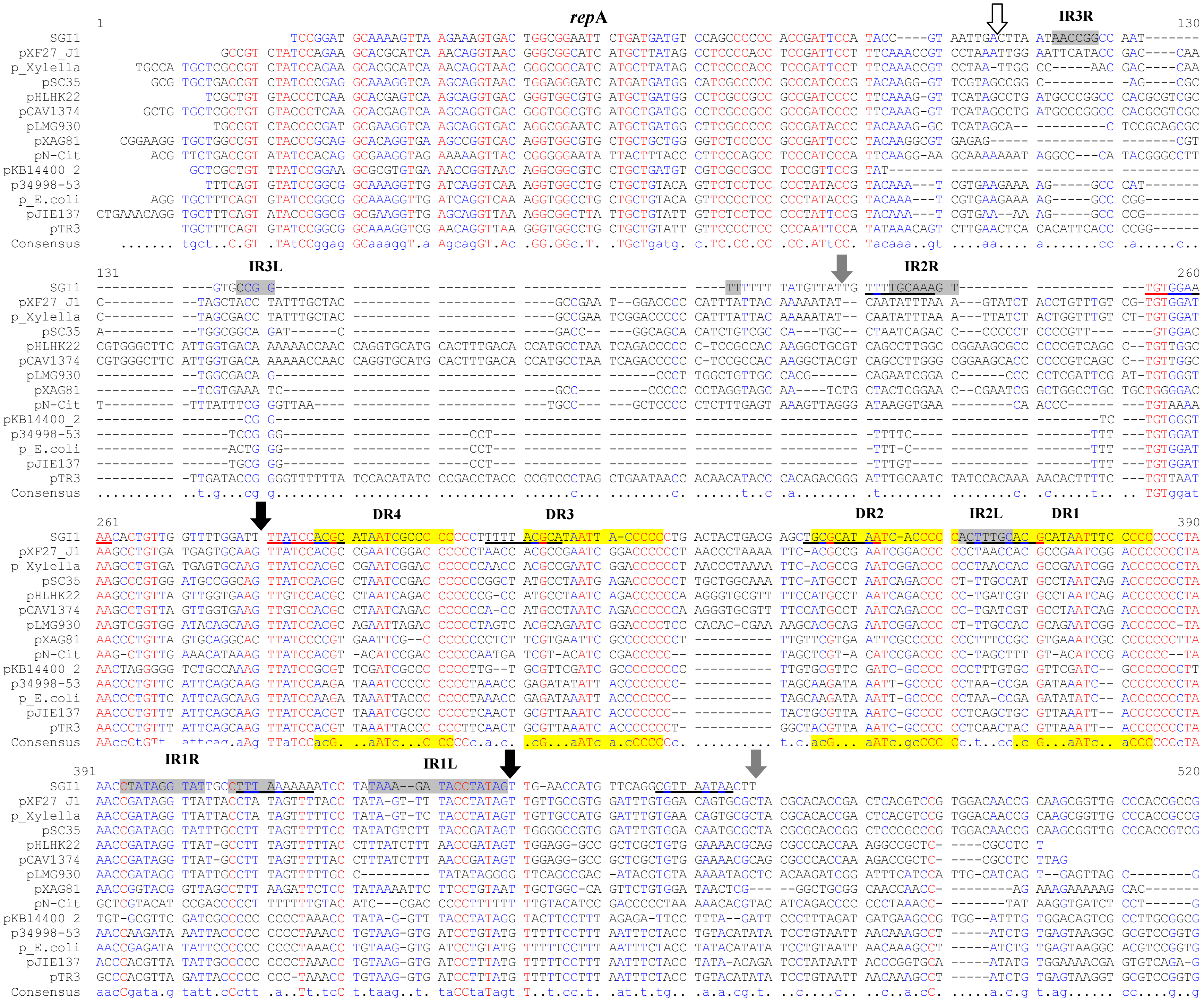

Suppl. fig. S2. Alignment of the 3' end of *repA* and the downstream noncoding region of SGI1 and selected IncN2/N3 plasmids. Open arrow: the end of *repA*$_{SGI1}$, grey arrows: ends of the fragment cloned in pMSZ1014, black arrows: ends of the fragment cloned in pMSZ1071, yellow boxes: DR1-4, grey boxes: IRs. Putative DnaA binding sites are underlined (potential DnaA-boxes were determined as sequences with ≤2 mismatches to the TT(A/T)TNCACA consensus of DnaA binding sites).
